# Deciphering genetic basis of phenylethanoid glycoside production through integrative multiomics analysis in *Callicarpa nudiflora*

**DOI:** 10.1093/hr/uhag203

**Published:** 2026-06-10

**Authors:** Fulai Yu, Mingju Yao, Mei Huang, Xunmeng Feng, Lanlan Zang, Donghua Hu, Lanping Guo, Shichao Sun, Xue Qiao, Li Wang

**Affiliations:** State Key Laboratory of Tropical Crop Breeding, Tropical Crops Genetic Resources Institute, Chinese Academy of Tropical Agricultural Sciences, Key Laboratory of Biology and Cultivation of Herb Medicine (Haikou), Ministry of Agriculture and Rural Affairs, Key Laboratory of Tropical Crops Germplasm Resource Genetic Improvement and Innovation of Hainan Province, Sanya/Haikou 572024/571101, China; State Key Laboratory of Natural and Biomimetic Drugs, School of Pharmaceutical Sciences, Peking University, Beijing 100191, China; State Key Laboratory of Tropical Crop Breeding, Tropical Crops Genetic Resources Institute, Chinese Academy of Tropical Agricultural Sciences, Key Laboratory of Biology and Cultivation of Herb Medicine (Haikou), Ministry of Agriculture and Rural Affairs, Key Laboratory of Tropical Crops Germplasm Resource Genetic Improvement and Innovation of Hainan Province, Sanya/Haikou 572024/571101, China; Shenzhen Branch, Guangdong Laboratory of Lingnan Modern Agriculture, Genome Analysis Laboratory of the Ministry of Agriculture and Rural Affairs, Agricultural Genomics Institute at Shenzhen, Chinese Academy of Agricultural Sciences, Shenzhen 518120, China; Shenzhen Branch, Guangdong Laboratory of Lingnan Modern Agriculture, Genome Analysis Laboratory of the Ministry of Agriculture and Rural Affairs, Agricultural Genomics Institute at Shenzhen, Chinese Academy of Agricultural Sciences, Shenzhen 518120, China; Shenzhen Branch, Guangdong Laboratory of Lingnan Modern Agriculture, Genome Analysis Laboratory of the Ministry of Agriculture and Rural Affairs, Agricultural Genomics Institute at Shenzhen, Chinese Academy of Agricultural Sciences, Shenzhen 518120, China; State Key Laboratory for Quality Ensurance and Sustainable Use of Dao-di Herbs, National Resource Center for Chinese Materia Medica, China Academy of Chinese Medical Sciences, Beijing 100700, China; Shenzhen Branch, Guangdong Laboratory of Lingnan Modern Agriculture, Genome Analysis Laboratory of the Ministry of Agriculture and Rural Affairs, Agricultural Genomics Institute at Shenzhen, Chinese Academy of Agricultural Sciences, Shenzhen 518120, China; State Key Laboratory of Natural and Biomimetic Drugs, School of Pharmaceutical Sciences, Peking University, Beijing 100191, China; Shenzhen Branch, Guangdong Laboratory of Lingnan Modern Agriculture, Genome Analysis Laboratory of the Ministry of Agriculture and Rural Affairs, Agricultural Genomics Institute at Shenzhen, Chinese Academy of Agricultural Sciences, Shenzhen 518120, China

## Abstract

*Callicarpa nudiflora* is a medicinal plant rich in phenylethanoid glycosides (PhGs), particularly verbascoside, but the genetic basis of PhG biosynthesis and natural variation in its content remain largely unexplored. Here, we assembled a chromosome-level genome of *C. nudiflora* (511 Mb, contig N50 = 12.78 Mb) with 39 317 annotated protein-coding genes and 99.5% Benchmarking Universal Single-Copy Orthologs completeness and quantified its bioactive compounds and gene expression across multiple tissues. Using the dataset, we elucidated the complete biosynthetic pathway of verbascoside and demonstrated multiple paralogous enzymes catalyzing one step, a functional redundancy that may contribute to the high verbascoside accumulation observed in this species. Population genomic analysis, together with quantification of verbascoside content of 95 samples grown in a common garden over three consecutive years, revealed that Southcentral Hainan was identified as the genetic diversity center and exhibited the highest content of verbascoside*.* Selective sweep analysis comparing high- and low-content individuals identified *CnUGT79G7*, a key glycosyltransferase in the verbascoside pathway, as being under positive selection. Two major haplotypes of *CnUGT79G7* were identified: Hap1, a high-expression haplotype prevalent in the top-geoherb region (77% frequency), and Hap2, a low-expression haplotype fixed in other populations. Although the two haplotypes encode proteins with similar catalytic activity, Hap1 is associated with significantly higher *CnUGT79G7* expression and elevated verbascoside content, suggesting that cis-regulatory variation underlies chemotypic differentiation. Collectively, this work identifies key molecular components for verbascoside biosynthesis, establishes a genomic and metabolic framework for understanding top-geoherb, and provides valuable resources for the conservation and molecular breeding of medicinal plants.

## Introduction

Phenylethanoid glycosides (PhGs) are plant-derived phenolic compounds known for their intricate structures [1, 2], which are composed of phenylethyl alcohol, caffeic acid, and glycosyl moieties. The central glycosyl group is usually glucose, while the outer glycosyl group could be glucose, rhamnose, apiose, xylose, arabinose, or galactose. Until now, nearly 600 PhGs have been identified from plants. The well-known and common types are the diglycoside PhGs (~38%), including verbascoside and cistanoside modified with glucose and rhamnose, and the trisaccharide PhGs (~43%), including forsythoside B and echinacoside modified with glucose, rhamnose or apiose [3, 4]. PhGs demonstrate a broad range of biological activities, including antibacterial, antivirus, anti-inflammatory, analgesic, neuroprotective, cardioprotective, antifatigue, hepatoprotective, kidney function, and immunomodulatory effects [4, 5]. Echinacoside and verbascoside are currently under clinical investigation for the treatment of generalized anxiety disorder and Immunoglobulin A nephropathy (National Clinical Trial (NCT): 03463018, 02662283), respectively [6]. Consequently, PhGs have demonstrated substantial potential for development of new drugs and health supplements [7, 8].

Through massive endeavors in the past 10 years, researchers have basically reached a consensus on the general PhG biosynthetic pathway (Fig. S1). The core pathway initiates with tyrosol glucosylation to salidroside, followed by coumaroylation (catalysed by acyltransferases like SiAT1) forming osmanthuside A, and subsequent 1,3-rhamnosylation (catalysed by glycosyltransferases like UGT79G7) yielding osmanthuside B. Network pathways are proposed for the conversion of osmanthuside B to verbascoside. One pathway involves two-step consecutive hydroxylation reactions of osmanthuside B to produce verbascoside [9]. Another pathway requires caffeoylation of salidroside, followed by the rhamnosylation step, and concluding with one-step hydroxylation to yield verbascoside [10]. Previous research has characterized specific cytochrome P450 monooxygenases (e.g. from the CYP98A family) responsible for hydroxylation steps in PhG biosynthesis in species such as *Ligustrum robustum* [9, 11] and *Cistanche tubulosa* [10]. Furthermore, it is well established that paralogous enzyme genes may retain ancestral functions [12], undergo neofunctionalization [13], or become pseudogenized [14]. When multiple gene copies retain the capacity to catalyze the same biochemical step, this results in enzymatic redundancy. Such redundancy has been proposed as a potential mechanism driving the high accumulation of certain secondary metabolites (SMs) by increasing overall metabolic flux. However, this hypothesis awaits further investigation across multiple plant lineages.

PhGs are mainly distributed in several angiosperm families, including Lamiaceae, Scrophulariaceae, Asteraceae, Oleaceae, and Orobanchaceae. Although the verbascoside biosynthetic pathway was elucidated in plants such as *L. robustum* [9, 11], *Forsythia suspensa* [6], and *C. tubulosa* [10], it remains rarely reported in the Lamiaceae family, a family rich in PhG-accumulating medicinal plants. It is unclear whether the pathway elucidated in other families is conserved in Lamiaceae or if lineage-specific variations exist.

*Callicarpa nudiflora*, a classic herbal medicine in Lamiaceae recorded in Chinese Pharmacopoeia (2025), is well known for the abundant diversity and content of PhGs, among which verbascoside, forsythoside B, and isoverbascoside are regarded as the main bioactive compounds [15, 16]. *Callicarpa nudiflora* has been used to subdue swelling and hemostasis in the tradition of ‘Li’ minor-nationality [17], and to treat bleeding in the respiratory and digestive tract and bacterial infection caused by excessive blood heat and toxin [18]. It widely grows in Guangdong, Guangxi, and Hainan province of China as well as Bangladesh, India, Malaysia, Myanmar, Singapore, Sri Lanka, and Vietnam. Our previous studies showed that the PhG content varied profoundly among *C. nudiflora* populations, with populations from the central Mountain region in Hainan regarded as the top-geoherbalism [19, 20]; i.e. herbs grown in certain native ranges demonstrate better quality and efficacy than those grown elsewhere [21]. However, the genetic basis underlying the natural variation in PhG content remains unexplored. Understanding whether this variation is driven by natural selection acting on certain enzyme genes or by regulatory differences affecting gene expression is a fundamental question in medicinal plant biology.

Advances in sequencing technology as well as comparative and population genomic tools have greatly facilitated the discovery of significant candidate genes for biosynthetic pathways [22]. Comparative genomic approaches have revealed key enzymes involved in the biosynthesis of hyoscyamine and scopolamine among *Brugmansia arborea*, *Anisodus tanguticus*, and *Mandragora caulescens* in the Solanaceae family [23], as well as arginine decarboxylase (ADC), uridine diphosphate glucosyltransferase (UGT), and serine carboxypeptidase-like (SCPL) acyltransferase enzymes involved in the biosynthesis of leonurine in Motherwort [24]. Population genetic analysis also helped unravel the genetic basis regulating SM content. For example, the selected genes in cannabis accessions for drug usage, compared to hemp, were the key genes involved in cannabinoid biosynthesis [25]. Taken together, comparative and population genomics algorithms are proven to be powerful tools for screening genes involved in SM biosynthesis and genetic basis of variation of SM content among populations of medicinal plants.

In this study, we have collected a total of 95 *C. nudiflora* individuals and planted live samples in the common garden set up in Danzhou, Hainan province (109.50°E, 19.52°N). Based on the well-established common garden, we de novo assembled a chromosome-level genome of a *C. nudiflora* accession with high verbascoside content (collected from Mt. Wuzhishan in Hainan; Fig. 1A). Widely targeted SMs and gene expression levels in the root, stem, leaf, and flower of *C. nudiflora* were determined. Whole-genome resequencing data of its populations spanning Hainan, Guangdong, and Guangxi Province, together with quantification of verbascoside of all samples across three consecutive years (2021–2023), were produced. With such a solid database, we aim to (i) uncover the genetic diversity and population differentiation of *C. nudiflora*; (ii) decipher the catalyzing enzymes in verbascoside biosynthetic pathway; and (iii) dissect genetic underpinnings of the difference of verbascoside content among populations.

**Figure 1:**
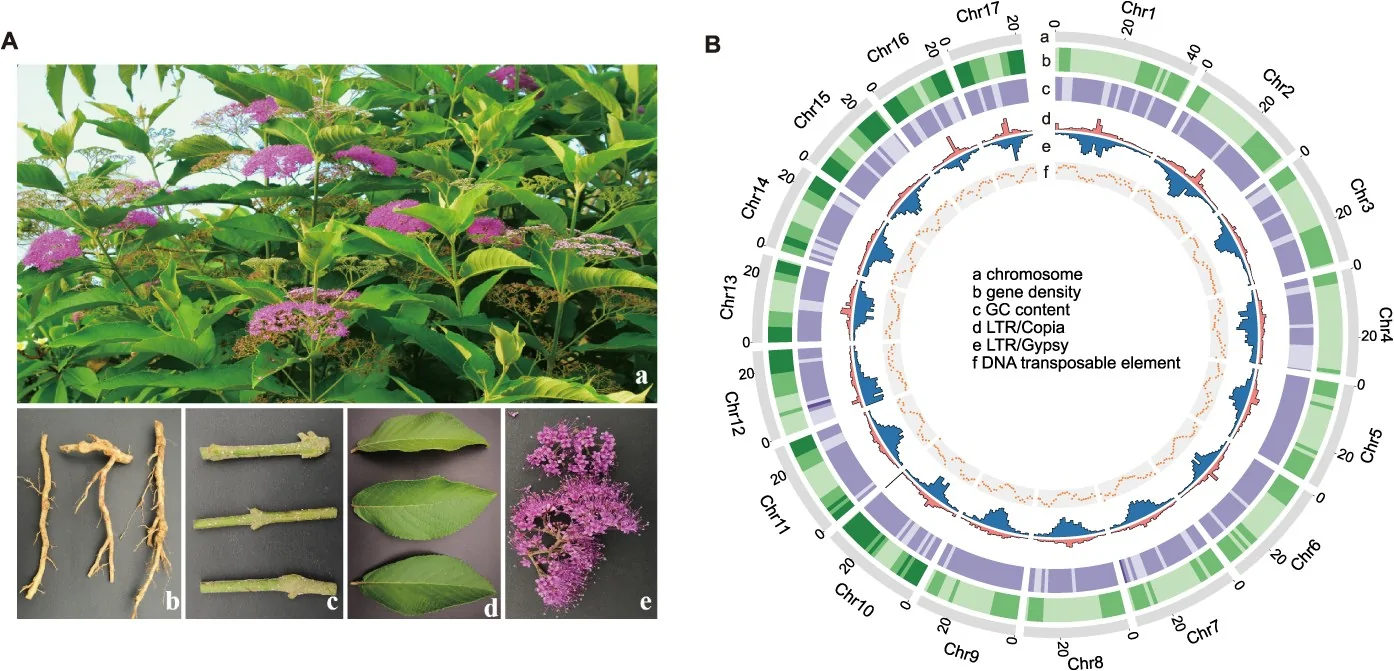
Images and genomic landscapes of *C. nudiflora*. (A) Images of the whole plant and its multiple tissues: (a) the whole plant of one flowering *C. nudiflora*, (b) roots, (c) stems, (d) leaves, and (e) flowers. (B) Circos plot of genomic characteristics of *C. nudiflora*. Circles from outside to inside: (a) chromosomes, (b) gene density, (c) GC content, (d) Copia LTR density, (e) Gypsy LTR density, and (f) DNA transposable element density. These density metrics were calculated with 500 kb non-overlapped sliding windows.

## Results

### Genome assembly and annotation of *C. nudiflora*

Fluorescence in situ hybridization (FISH) analysis revealed 17 pairs of homologous chromosomes in metaphase root tip cells, confirming that *C. nudiflora* is a diploid species with 2*n* = 2*x* = 34 (Fig. 1A; Fig. S2). Based on K-mer analysis of short-read sequences (Fig. S3), the genome size of *C. nudiflora* was estimated to be approximately 516 Mb, with about 0.09% heterozygosity, which is similar with the genome size of the congeneric species *Callicarpa americana* [26]. Based on this estimated genome size, a total of 139.87 Gb (~268× coverage) PacBio continuous long reads (CLR) were generated. Assembly of the CLR reads resulted in a 511.19-Mb genome with a contig N50 of 12.78 Mb, which is approximately twice that of *C. americana* [26]*.* We further scaffolded the contigs into pseudochromosomes using a high-throughput chromosome conformation capture (Hi-C) interaction matrix (Fig. S3). A total of 96.79% of the assembled contigs were anchored onto 17 pseudochromosomes, yielding a scaffold N50 of 30.54 Mb (Fig. 1B; Table S1). Compared with the published *C. americana* genome, the overall quality of our *C. nudiflora* assembly was higher (Table S1). Benchmarking Universal Single-Copy Orthologs (BUSCO) analysis indicated that 99.50% of the core viridiplantae orthologous genes were completely present in the *C. nudiflora* assembly, compared to 98.80% in *C. americana* (Fig. S4). The assembled genome also showed a long terminal repeat assembly index (LAI) of 12.84, indicative of reference-quality assembly, and higher than the LAI of 8.68 reported for *C. americana* (Table S1).

By integrating *ab initio* prediction, homology-based method, and RNA sequencing (RNA-seq) evidence, we identified 39 317 protein-coding genes in the *C. nudiflora* genome (Table S1), with 88.89% functionally annotated. The average gene and coding sequence length were 3301.26 and 1141.22 bp, respectively. Furthermore, transposable elements (TEs) accounted for 252.78 Mb (49.45%) of the genome. Among these, the two major categories of long terminal repeats (LTRs), LTR/Gypsy and LTR/Copia, represented 21.96% and 15.64% of the genome, respectively. Consistent with the high-quality genome assembly, the protein-level BUSCO assessment showed 97.20% completeness in the core viridiplantae orthologous gene set (Fig. S5), supporting the high quality of the *C. nudiflora* genome annotation.

### Phylogenetic and comparative genomic analysis of *C. nudiflora*

To investigate the evolutionary history of *C. nudiflora*, we reconstructed a phylogenetic tree using 658 single-copy orthologous genes shared among *C. nudiflora*, *C. americana*, *Olea europaea*, *Thymus quinquecostatus*, *Salvia bowleyana*, *Salvia hispanica*, *Scutellaria baicalensis*, *Tectona grandis*, and two outgroups (*Vitis vinifera* and *Ilex asprella*). Divergence times were estimated with the calibration point set at the split time between *O. europaea* and *V. vinifera* [*27**]*. Within Lamiales, the divergence between Oleaceae and Lamiaceae occurred at ~112.34 MYA. The diversification within Lamiaceae was dated to ~69.64 MYA (95% highest posterior density [HPD]: 62.17–84.11 MYA), consistent with a previous report [28]. The split time between the Nepetoideae (*S. hispanica*, *S. bowleyana*, *T. quinquecostatus*) and Callicarpoideae (*C. americana*, *C. nudiflora*) subfamilies was ~56.74 MYA (95% HPD: 47.98–71.58 MYA). The divergence time between *C. nudiflora* and *C. americana* was estimated as ~12.71 MYA (95% HPD: 6.63–22.31 MYA) (Fig. 2A).

**Figure 2:**
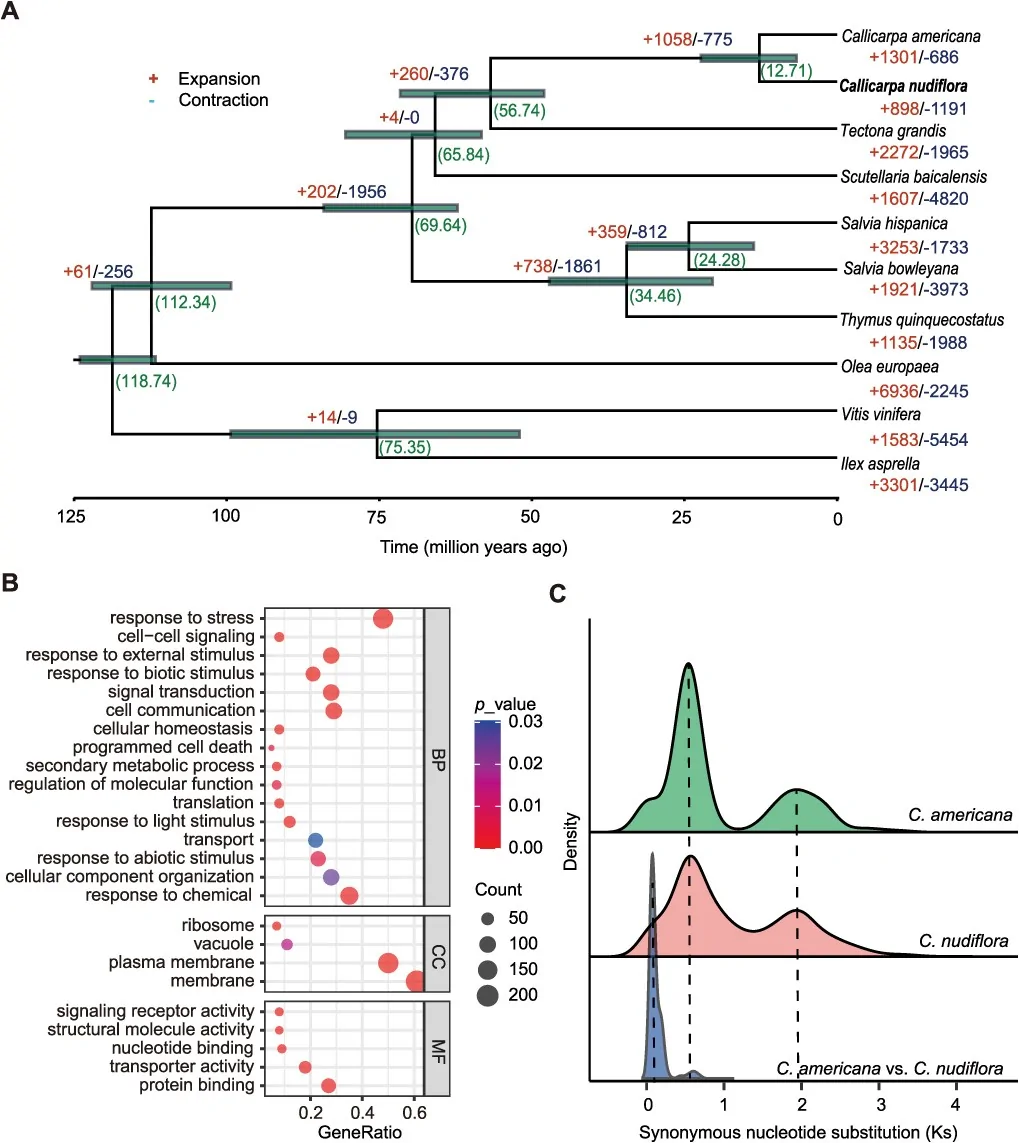
Phylogeny, time tree, gene family expansion and contraction, and WGD analyses in *C. nudiflora*. (A) Phylogenetic tree illustrating divergence times among 10 Lamiaceae species and two outgroups. Green bars indicted 95% confidence intervals of the estimated divergence time. ‘+’ and ‘-’ signs showed the numbers of gene families that underwent expansions or contractions. (B) Functional enrichment of expanded gene families in *C. nudiflora*. (C) Ks distribution from paralogs within *C. americana*, paralogs within *C. nudiflora*, and orthologs between the two species.

By analyzing gene family evolution across the nine species in the phylogenetic tree, we explored lineage-specific and expanded/contracted gene families in *C. nudiflora* and the common ancestor of the *Callicarpa* genus. Four-hundred and eighty seven families (1948 genes) were unique to *C. nudiflora* among the total of 9123 families (36 520 genes) (Fig. S6). These species-specific families were significantly enriched (corrected *P* < .05) in functions related to stress responses, abiotic stimuli, and chemical responses (Table S2), suggesting enhanced adaptive capacity. Relative to the most recent common ancestor (MRCA), 898 gene families were expanded and 1191 contracted in *C. nudiflora* (Fig. 2A). Expanded families were enriched in biological processes associated with environmental adaptation, including responses to stress, external and biotic stimuli, light, chemicals, abiotic stress, and secondary metabolism (Fig. 2B). Contracted families were also enriched in biotic stress responses and secondary metabolism (Table S3), indicating possible fine-tuning of the gene repertoire for diverse environmental challenges. In the *Callicarpa* genus, expanded families were similarly enriched in secondary metabolism and stress/stimulus responses (Table S4), suggesting a shared adaptive strategy across the genus.

Whole-genome duplication (WGD) is a major driver of flowering plant evolution [29]. To identify potential WGD events, we examined the distribution of synonymous substitution rates (Ks) for paralogous gene pairs in *C. nudiflora* and *C. americana*. Both species showed two distinct Ks peaks (~0.67 and ~1.9), indicating shared WGDs (Fig. 2C). The Ks peak for orthologous genes between *C. nudiflora* and *C. americana* (~0.1) suggests that these two WGDs occurred before their divergence (Fig. 2C). The more recent WGD in *C. nudiflora* was dated to ~50 MYA, using a synonymous substitution rate of 6.59 × 10^−9^ substitutions/site/year estimated in lavender [28]. This event predates the split of *C. americana* and *C. nudiflora,* implying that the WGD possibly occurred in the common ancestor of *Callicarpa*. The older WGD (Ks ≈ 1.9) was dated to ~144 MYA and corresponds to the γ whole-genome triplication shared by core eudicots [30].

### Metabolite profiling of *C. nudiflora* reveals its bioactive compounds

*Callicarpa nudiflora*, an important tropical medicinal herb, is rich in SMs [17]. Using an UPLC-MS/MS metabolomics approach, we identified 727 chemical constituents across four tissues (root, stem, leaf, and flower). Flowers contained the largest diversity of SMs (675 compounds), whereas roots contained the smallest diversity of SMs (Fig. 3A). Principal component analysis (PCA) revealed clear metabolic differentiation among the four tissues (Fig. 3B). Among the identified metabolites, 348 were detected in all tissues. Organic acids and derivatives (17.5%), amino acids and derivatives (14.0%), lipids (13.7%), and nucleotides and derivatives (10.3%) were the most abundant classes, whereas phenolic acids (0.2%), flavonoids (0.2%), and isoflavones (0.2%) were the least abundant. Several SMs were tissue-specific, including 54 in flowers, 16 in leaves, and two in roots (Fig. 3C).

**Figure 3:**
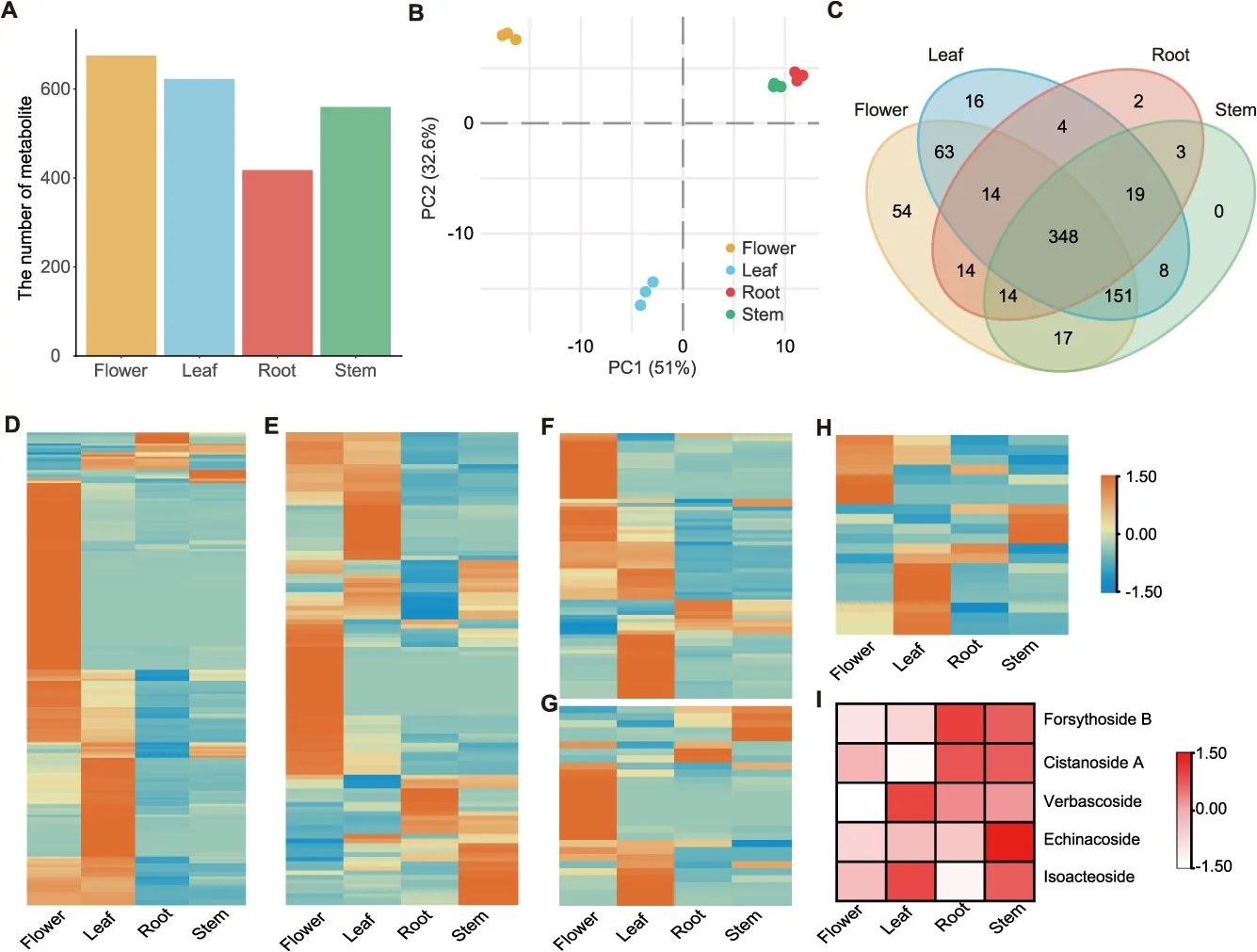
Metabolomic profiling of *C. nudiflora*. (A) Numbers of the quantified secondary metabolites in different tissues. (B) Principal component analysis of widely targeted metabolites in different tissues of *C. nudiflora*. (C) Statistics of common and tissue-specific secondary metabolites, along with major substances, in different tissues. (D–H) Diversity analysis of five major classes of bioactive compounds, : (D) flavonoids, (E) phenylpropanoids, (F) lipids, (G) alkaloids, and (H) terpenoids,in four tissues of *C. nudiflora*. (I) Distribution of PhG contents among four tissues of *C. nudiflora*.

To identify potential bioactive substances for disease treatment, bioactive components were classified into five major categories: flavonoids (214), phenylpropanoids (103), lipids (68), alkaloids (28), and terpenoids (20) (Table S5). These compounds were predominantly enriched in flowers and leaves (Fig. 3D–H). Within phenylpropanoids, six PhGs were detected, including verbascoside, a key medicinal constituent of *C. nudiflora* [31, 32]. These PhGs showed distinct tissue-specific patterns (Fig. 3I): verbascoside was abundant in stems and leaves; cistanoside A and forsythoside B were mainly detected in roots and stems; isoverbascoside was enriched in stems and leaves; both 2′-acetylacteoside and echinacoside were mainly accumulated in stems.

### The elucidation of biosynthetic pathway for verbascoside

The biosynthetic pathway of verbascoside in *L. robustum* has been elucidated [9, 11]. Key enzymes include the glycosyltransferase LrUGT85AF, which converts tyrosol to salidroside; the hydroxycinnamoyl-CoA:salidroside hydroxycinnamoyltransferase LrAT1, which acylates salidroside to form osmanthuside A; the osmanthuside A 1,3-rhamnosyltransferase LrUGT79G7, which converts osmanthuside A to osmanthuside B; and the osmanthuside B 3,3′′-hydroxylase LrCYP98A194, which produces verbascoside from osmanthuside B. These four enzymes were used as queries in BLAST searches against the *C. nudiflora* protein database. We identified homologous genes with >70% protein sequence identity, including *CnUGT85AFs* (*Cn06G0864*, *Cn06G0865*), *CnATs* (*Cn05G1944*, *Cn05G1945*), *CnUGT79G7* (*Cn02G0899*), and *CnCYP98As* (*Cn05G2248*, *Cn16G0232*).

To verify the functions of the seven candidate genes, we conducted *in vitro* enzyme activity assays. CnUGT85AFs, CnATs, and CnUGT79G7 were expressed in *Escherichia coli* using the pET-28a system, and CnCYP98As were expressed in the *Saccharomyces cerevisiae* WAT11 strain (Table S6). Two CnUGT85AF enzymes catalysed the glucosylation of tyrosol (i) to form salidroside (ii) (Fig. 4A; Fig. S7). CnATs then converted salidroside into osmanthuside A (iii) (Fig. 4B), with MS^2^ analysis showing a diagnostic fragment ion at m/z 145.03, confirming coumaroylation on the glucoside core (Fig. S8). CnUGT79G7 catalyzed the 3′-*O*-rhamnosylation of osmanthuside A to yield osmanthuside B (iv) (Fig. 4C), as indicated by a + 146 Da mass shift (Fig. S9). CnCYP98As converted osmanthuside B into more polar products, including verbascoside (5, m/z 623.20), identified by comparison with a reference standard (Fig. 4C). An intermediate (m/z 607.20) containing the coumaroyl group (m/z 145.03) was also detected (Fig. S10). These results elucidated the verbascoside biosynthetic pathway in *C. nudiflora*.

**Figure 4:**
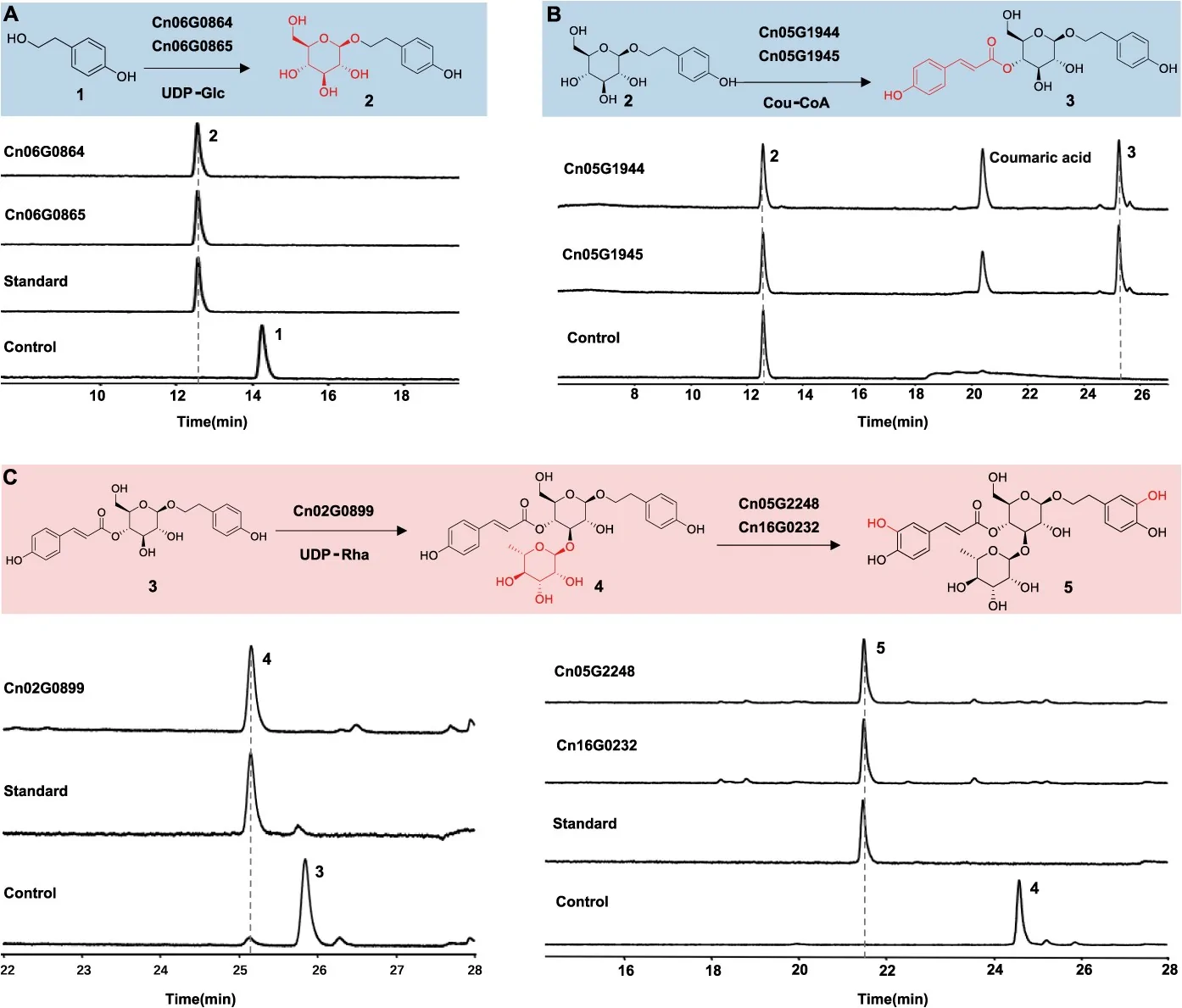
Complete biosynthetic pathway of the phenylethanoid glycoside verbascoside in *C. nudiflora*. (A) Functional characterization of *Cn06G0864* and *Cn06G0865* using compound 1 as the substrate. (B) Functional characterization of *Cn05G1944* and *Cn05G1945* using compound 2 as the substrate. (C) Functional characterization of *Cn02G0899* using compound 3 as the substrate and functional characterization of *Cn05G2248* and *Cn16G0232* using compound 4 as the substrate.

To trace the evolutionary origin of the functional redundancy in *C. nudiflora*, we performed a comparative synteny analysis across *C. nudiflora*, *C. americana*, *S. baicalensis*, and *S. bowleyana* (Fig. S11). It showed that the seven functional catalyzing enzyme homologs in *C. nudiflora* are located within highly conserved syntenic blocks shared among these Lamiales species, however, the two functional genes catalyzing one step have two corresponding syntenic genes in *C. americana*, but only one syntenic gene in *S. baicalensis* and *S. bowleyana*. These results indicated that the redundant copies may have arisen from the common ancestor of *Callicarpa* genus via some modes of duplication events (Fig. S11A–D). To further validate this, we examined their expression patterns across four tissues in *C. nudiflora*. These seven functional genes exhibited predominant expression in roots and leaves (Fig. S12), and demonstrated strong positive correlations between the expression levels of the seven genes and verbascoside content, with correlation coefficients (*r*) ranging from 0.62 (for *Cn06G0864*) to 0.96 (for *Cn16G0232*). This consistent expression-accumulation pattern suggests that these redundant genes might have been maintained to collectively enhance metabolic flux through a dosage effect. Such a mechanism likely ensures a robust supply of enzymes to support the high-level synthesis of verbascoside in *C. nudiflora*, providing both pathway robustness and physiological efficiency.

### Population structure and genetic diversity of *C. nudiflora*

We generated 1584 Gb of clean whole-genome resequencing data from 95 *C. nudiflora* samples collected in Hainan, Guangdong, and Guangxi, China (Fig. 5A). The average sequencing depth was 32.73X (range: 18.69–52.44X), with mapping rates between 92.17% and 99.58% (Table S4). A total of 14 112 559 high-quality single nucleotide polymorphisms (SNPs) were identified for population genetic analyses.

**Figure 5:**
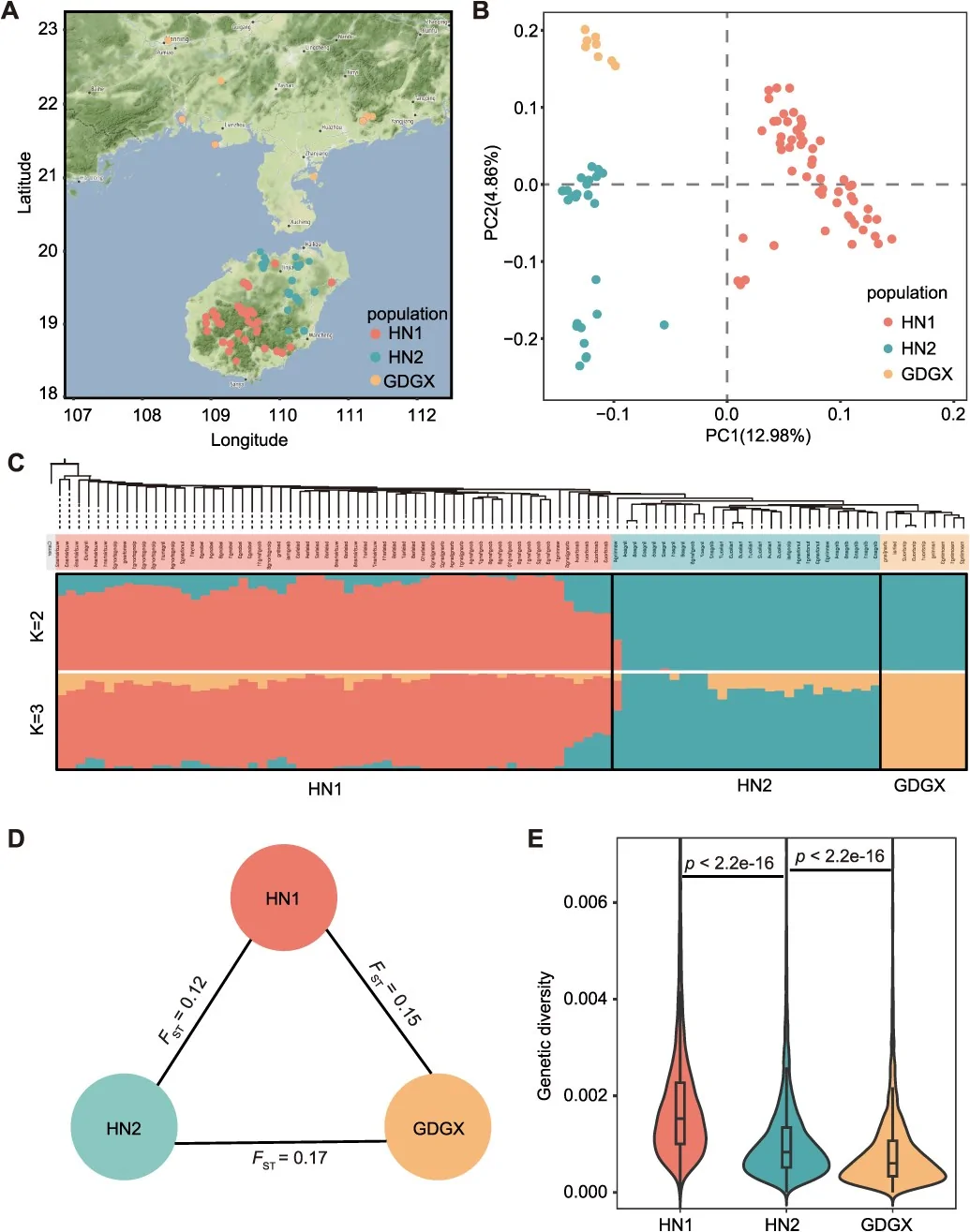
Distribution, population structure, genetic differentiation, and genetic diversity of *C. nudiflora* populations. (A) Geographic distribution of the resequenced accessions of *C. nudiflora*. (B) Principal component analysis of 95 *C. nudiflora* accessions. (C) Phylogeny and population structure analyses of *C. nudiflora* accessions. (D) Genetic divergence among *C. nudiflora* subpopulations estimated with *F*_ST_. Summary statistics were calculated in 100-kb sliding windows with 10-kb step size. (E) Boxplot of genetic diversity (π) among populations. Summary statistics were calculated in 100-kb sliding windows with 10-kb step size. *P* values were calculated with two-sided Wilcoxon test.

We assessed the population structure of *C. nudiflora* using three complementary methods. PCA of 696 152 filtered SNPs (minor allele frequency > 0.05, missing rate <10%, LD-pruned) showed that individuals from southwestern Hainan (HN1; 58 samples) formed a distinct cluster along PC1, separate from northern Hainan (HN2; 28 samples) and Guangdong-Guangxi (GDGX; nine samples) groups (Fig. 5B). Phylogenetic tree reconstruction and model-based Admixture analysis confirmed the population structure. At *K* = 3, the non-HN1 samples were further divided into HN2 and GDGX clusters (Fig. 5C).

*F*_ST_ was calculated to evaluate genetic differentiation between pairwise populations of *C. nudiflora.* It revealed that the genetic divergence between HN1/HN2 and GDGX was approximately 0.15 and 0.17, respectively, whereas the genetic divergence between HN1 and HN2 was 0.12 (Fig. 5D). HN1 exhibited the highest nucleotide diversity (π) (1.9 × 10^−3^), followed by HN2 (1.1 × 10^−3^), and the lowest in GDGX (8.6 × 10^−4^) (Fig. 5E). Heterozygosity followed the same pattern, with HN1 showing the highest and GDGX demonstrating the lowest levels (Fig. S13). These findings indicate that southwestern Hainan (HN1) is likely the genetic diversity center of *C. nudiflora* in China.

### An UGT enzyme under positive selection explains abundance difference of verbascoside among *C. nudiflora* populations

HN1, encompassing Mt. Wuzhishan, is considered the top-geoherb region of *C. nudiflora* and was expected to show high verbascoside abundance. Indeed, verbascoside levels in HN1 were significantly higher than in HN2 and GDGX, with no significant difference between the latter two groups (Fig. 6A). To elucidate the genetic basis determining verbascoside content in *C. nudiflora*, we compared *F*_ST_ and π-ratio between the top 15 high-content and 15 low-content samples and identified SNPs with the top 5% *F*_ST_ and π-ratio values as under selection (*F*_ST_ ≥ 0.36 and π-ratio ≥ 1.27). These SNPs were assigned to 4728 candidate genes (see Methods), which included the key biosynthetic gene *CnUGT79G7* (Fig. 6B). *CnUGT79G7* (chr2: 10808438–10 810 438 bp) contained five SNPs in the promoter region and two nonsynonymous SNPs in the exon region (Fig. 6C). These seven SNPs defined two major haplotypes. Individuals with Hap1 had significantly higher verbascoside content than those with Hap2 (*P* = 0.014, Wilcoxon test), indicating Hap1 as the favorable haplotype (Fig. 6D). Hap1 predominated in HN1 (77%), whereas all individuals in HN2 and GDGX carried Hap2 (Fig. 6E), suggesting positive selection of Hap1 in HN1 contributed to its elevated verbascoside levels.

**Figure 6:**
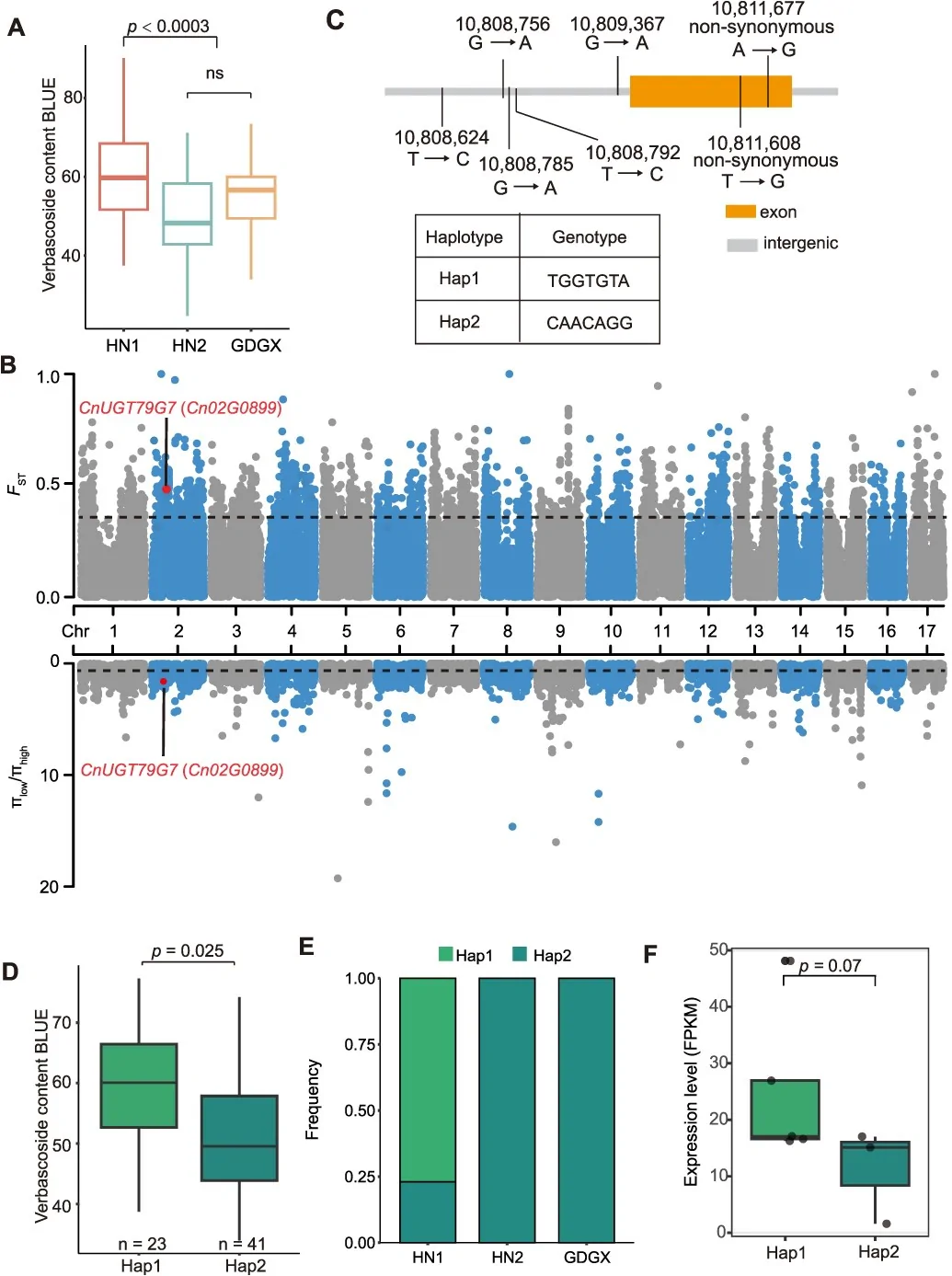
Genetic underpinnings of verbascoside content. (A) Verbascoside content in three subpopulations of *C. nudiflora*. (B) Manhattan plot of genetic diversity (π) ratio and genetic differentiation (*F*_ST_) between 15 individuals with the lowest and highest verbascoside content, respectively. (C) Gene structure and haplotype analyses of *Cn02G0899*, encoding an UGT enzyme converting osmanthuside A into osmanthuside B. (D) Boxplots of verbascoside content in individuals harboring the two haplotypes. In each plot, the center line indicates the median, the box limits denote the upper and lower quartiles, and the whiskers represent 1.5 times of the interquartile ranges; *n* indicates the sample size. (E) Haplotype frequency of *Cn02G0899* in the three populations. (F) Expression levels of *Cn02G0899* in accessions carrying the two haplotypes. *P* values were calculated with two-sided Wilcoxon test.

To compare the catalyzing activity of the two haplotypes, we performed *in vitro* enzyme assays of the two CnUGT79G7 protein variants. Both Hap1 and Hap2 showed similar catalytic activity toward the substrate (Fig. S14). However, expression analysis revealed that expression levels of *CnUGT79G7* were higher in Hap1 homozygous samples than in Hap2 homozygous samples (Fig. 6F). This expression difference, together with the observation that the two haplotypes differ by five SNPs in the promoter region and two nonsynonymous SNPs in the coding region (Fig. 6C), indicates that the difference of verbascoside content between samples with different haplotypes was likely caused by transcriptional regulation rather than altered enzyme activity. The correlation between promoter variants and gene expression points to potential cis-regulatory differences, though direct functional validation will be required to establish the causal relationship.

## Discussion

In this study, based on a de novo assembled chromosome-level genome of *C. nudiflora*, whole-genome-resequencing data of its populations spanning Hainan, Guangdong, and Guangxi Province, together with quantification of verbascoside of all samples planted in a common garden across three consecutive years, we infer that South central Hainan (including Mt. Wuzhishan) is likely its diversity center and exhibits the highest content of verbascoside, which is probably correlated with positive selection of one favorable haplotype of an UGT gene that is involved in the verbascoside biosynthesis.

*Callicarpa nudiflora* remains an underappreciated medicinal plant despite its high potential for research and exploitation. First, unlike most traditional Chinese medicines (TCM) used in multiherb prescriptions, *C. nudiflora* can be applied as a monotherapy for the treatment of respiratory and digestive tract hemorrhages, as well as bacterial infections caused by ‘blood heat’ and pathogenic toxins [18]. This unique property highlights its value for investigating pharmacological mechanisms and elucidating the biosynthetic pathways of its SMs. Second, as a traditional medicine of the Li ethnic group, *C. nudiflora* provides a representative case for safeguarding and transmitting indigenous medicinal knowledge, and for studying the interactions between medicinal resources and human populations. Third, while most medicinal plants in the Lamiaceae family produce terpenoids as their primary bioactive compounds—as exemplified by lavender (*Lavandula angustifolia*) [28] and rosemary (*Rosmarinus officinalis*) [33]—*C. nudiflora* is unusual in that its hemostatic effect is primarily attributed to PhGs [31]. PhGs are widely distributed among dicotyledonous plants, including those in the Lamiaceae, Scrophulariaceae, Asteraceae, Oleaceae, and Orobanchaceae families. Several medicinal herbs, such as Cistanche spp., Rehmannia spp., *Ligustrum lucidum*, *F. suspensa*, and *Plantago asiatica*, are rich in PhGs. Collectively, these features poise *C. nudiflora* as a key species for investigating the evolution of PhG biosynthetic pathways among angiosperm families.

Comparative analysis of the verbascoside biosynthetic pathway in *C. nudiflora* and *L. robustum* reveals fundamental sequence conservation of the catalyzing enzymes. However, a notable distinction lies in the functional redundancy of catalyzing enzymes. Distinct from one enzyme acting on one reaction step, multiple enzymes catalyze one reaction step in *C. nudiflora*, except for the rhamnosylation reaction. This multienzyme redundancy may contribute to the substantially high verbascoside accumulation observed in *C. nudiflora,* compared with other angiosperm species containing PhGs [34] (Fig. S15). Likewise, in *Artemisia annua*, multiple copies of amorpha-4,11-diene synthase contribute to artemisinin production through dosage effects [35]. Our study provides empirical support for the hypothesis that gene duplication and retention can drive metabolic flux, which may represent a general strategy by which medicinal plants achieve high yields of pharmacologically active compounds. Our functional assays confirm that CYP98A enzymes catalyze consecutive hydroxylation reactions on the tyrosol and coumaroyl moieties, respectively, converting osmanthuside B directly into verbascoside. Nevertheless, the possibility of alternative hydroxylation routes in *C. nudiflora* remains to be explored. Specifically, it is unclear whether distinct CYP450 enzymes separately catalyze single hydroxylation steps on the tyrosol and coumaroyl groups. Additionally, the potential involvement of polyphenol oxidases (PPOs), which possess broad substrate promiscuity, in these hydroxylation reactions warrants further investigation [6, 9–11]. Importantly, the catalytic components involved in verbascoside biosynthesis identified from *C. nudiflora* exhibit exceptionally high *in vitro* activity. Such catalytic efficiency holds considerable promise for optimizing microbial heterologous production systems to achieve high verbascoside yields.

Population genetics combined with metabolite content analysis provides critical insights into the genetic mechanisms underlying the top-geoherbalism of medicinal plants, offering a robust framework for understanding their genetic adaptation and pharmacological efficacy [22]. Using selective sweep analysis, this study identified *CnUGT79*, a key enzyme gene in the verbascoside biosynthetic pathway, to be under positive selection. The high-expression haplotype was found to be prevalent in the top-geoherb region, the Southcentral Mountain area of Hainan. Although *in vitro* assays revealed no significant differences in catalytic activity between the two haplotypes, their distinct expression levels suggest that gene expression regulation, rather than enzymatic function, may be a driver of elevated verbascoside content in the top-geoherb region. The presence of five SNPs in the promoter region of *CnUGT79G7* that differentiate the two haplotypes points to potential cis-regulatory variation as a mechanism underlying the observed expression differences. However, we acknowledge that the current study establishes correlation rather than causation between these promoter variants and transcriptional activity. Direct experimental validation—such as promoter-reporter assays comparing the transcriptional activity of Hap1 and Hap2 promoter sequences in homologous or heterologous systems—will be necessary to confirm whether these SNPs directly affect transcription factor binding or promoter strength. Such functional characterization represents an important direction for future research to fully elucidate the molecular basis of the regulatory variation identified in this study.

Our multiomics approach—integrating chromosome-level genome assembly, tissue-specific metabolomics, functional enzyme characterization, and population genomics—aligns with a growing trend in medicinal plant research toward comprehensive systems-level analysis. Similar integrative strategies have proven powerful in other species. For instance, in *Cannabis sativa*, combined genomic and metabolomic analyses identified key enzymes under selection in drug-type accessions compared to fiber-type hemp [36, 37]. Likewise, population genomic studies in *Panax ginseng* have uncovered selection signatures in genes involved in ginsenoside biosynthesis, providing insights into the genetic basis of chemotype variation across cultivation regions [38, 39]. Our study extends this paradigm to *C. nudiflora*, demonstrating that the integration of common garden experiments with population genomics can minimize environmental variation and lend confidence to our conclusion that the observed content differences have a genetic basis. This integrative framework advances our understanding of the molecular mechanisms driving top-geoherbalism characteristics and holds significant implications for the conservation and breeding of medicinal plants with enhanced pharmacological properties.

Looking across medicinal plant studies, our work contributes to an emerging framework for understanding top-geoherbalism: diversity centers often harbor unique genetic variants in biosynthetic pathway genes, which are likely under selection; these variants possibly affect gene expression; and enzyme redundancy arising from gene duplication may predispose certain lineages to high metabolite accumulation. As more medicinal plant genomes become available and population-level resequencing studies accumulate, testing the generality of these patterns across diverse plant lineages and compound classes will be worthy of further investigation.

## Materials and methods

### Genome sequencing, assembly, and annotation

The young leaves of *C. nudiflora* from a single tree were used to extract high-quality genomic DNA for genome sequencing. We sequenced *C. nudiflora* genome using a combination of sequencing technologies, including long-read sequencing from Pacific Biosciences (PacBio), Illumina short-read sequencing, and chromosome conformation capture using Hi-C sequencing. A 50-μg amount of high-quality DNA was taken to construct SMRTbell libraries and sequenced in PacBio Sequel II platform with ‘Continuous Long Read (CLR) Sequencing’ mode. For Illumina short-read sequencing, DNeasy Plant Mini Kit (Qiagen, United States) was used to extract DNA, constructed library according to the manual of Nextera XT DNA Library Prep Kit (Illumina, Inc., San Diego, CA, USA), and sequenced on the Illumina NovaSeq 6000 platform. Hi-C library was constructed from the fresh leaves and then also sequenced on the Illumina NovaSeq 6000 platform.

PacBio CLR subreads were first self-corrected by CANU v2.0 [40]. After trimming the corrected subreads, the assembly was performed using ‘canu -assemble.’ Subsequently, Purge_haplotigs program v1.1.2 was employed to filter redundant contigs from the primary assembly. The resultant contigs underwent one round of alignment with PacBio CLR subreads using Racon v1.4.3 [41], and two rounds of alignment with Illumina short reads using NextPolish v1.2.0 [42], respectively. The Hi-C Illumina reads obtained from *C. nudiflora* were aligned to the corresponding contig sets using Juicer v1.6 [43]. Subsequently, scaffolding was carried out with 3D-DNA v180922 [44]. Manual correction of the connections was performed using Juicebox Assembly Tools v1.22 [45].

The Extensive de novo TE Annotator (EDTA v1.9.4) pipeline [46] was used to identify TE in the *C. nudiflora* genome, and the MAKER (v2.31.10) pipeline [47] was applied to predict the coding gene structure from *ab* initio predictions, homolog proteins, and transcriptome data. Briefly, transcript evidence was provided through a de novo assembled transcriptome created with TRINITY v2.11.0 [48], and a reference-based assembly generated by StringTie v2.1.4 [49]. The proteome data of *V. vinifera* [50], *I. asprella* [51], *O. europaea* [52], *T. quinquecostatus* [53], *S. bowleyana* [54], *S. hispanica* [55], *S. baicalensis* [56], and *C. americana* [26] as the protein evidence. The first round of MAKER incorporated transcript and protein evidence. AUGUSTUS v3.3.3 [57] and GENEMARK v4.69_lic [58] were trained using BRAKER2 v2.1.5, which utilized transcript data obtained from aligned RNA-Seq bam files generated by HISAT 2 v2.2.1 [59]. SNAP v2006-07-28 [60] was trained under MAKER with two iterations. In the second round of MAKER, the results from AUGUSTUS, GENEMARK, and SNAP were fed into MAKER again along with all other data to produce synthesized gene models.

### Comparative genomic analyses

The full amino acid sequences of *C. nudiflora*, *C. americana*, and the other eight species were aligned to orthologous groups using OrthoFinder v2.2.1 [61] with default settings. Single-copy genes were used to construct phylogenetic tree using the RAxML v8.2.12 [62] with PROTGAMMAJTT model and 1000 replicates. The divergence time among 10 plants was predicted using MCMCTree program in PAML v4.4 [63] based on the estimated divergence time *O. europaea*–*V. vinifera* (111–124 MYA) and *O. europaea*–*T. quinquecostatus* (64–109 MYA) in Timetree [64]. According to the phylogenetic analysis and divergence time, expansion and contraction of gene family were identified using CAFE v5.0.0 [65]. WGDI v0.6.5 [65] was used to detect the WGD events in Lamiales. GO enrichments were performed by ClusterProfiler v4.6.0 [66] for those significantly expanded or contracted gene families.

### Resequencing and variant calling

A total of 95 *C. nudiflora* samples were collected in 2016–2018, and planted at the common garden of tropical medicinal plants (Danzhou), Ministry of Agriculture and Rural Affairs, P.R. China. Genomic DNA was extracted following standard protocol for sequencing library construction with insert sizes of 150 bp and then were sequenced with the DNBSEQ-T7 platform.

Paired-end sequencing reads were mapped to the *C. nudiflora* genome using BWA-MEM 0.7.17-r1188 [67] with default parameters, and marked PCR duplicates using Picard v2.18.23 (https://broadinstitute.github.io/picard/). Samtools was used to generate sorted bam files [68]. GATK v4.2 [69] was used to call SNPs and hard filter with the following parameters: ‘QD < 2.0 || QUAL <30.0 || MQ < 40.0 || FS > 60.0 || SOR > 3.0 || MQRankSum < −12.5 || ReadPosRankSum < −8.0’, and only keep biallelic SNPs for further analysis.

### Phylogenetic tree, population genetic diversity, and structure analysis

To infer the basal group of *C. nudiflora*, we built a neighbor-joining tree based on filtering SNPs (minor allele frequency ≥ 0.05, missing data ≤10%, and minimum distance between two SNPs ≥2 kb) using VCF2Dis v.1.42 (https://github.com/BGI-shenzhen/VCF2Dis). PCA was performed with plink v1.90b6.18 [70], and the first two eigenvectors were plotted in 2D. Admixture v1.3.0 [71] software was used to infer the population structure with the likelihood of ancestral kinship (*K*) value from 2 to 3. Linkage disequilibrium was analyzed using PopLDdecay v3.41 [72] for the three groups, and LD decay was calculated on the basis of the *r^2^* value and the corresponding distance between two SNPs. VCFtools v0.1.16 [73] was used to detect nucleotide diversity (*π*) for the HN1, HN2, and GDGX populations with the following parameters: 100-kb sliding window and 10-kb step size.

### Screening for selective sweep

Nucleotide diversity (π) and *F*st are the two most commonly used parameters for detecting selective signatures between populations. For the analysis, 15 samples with the highest and 15 samples with the lowest verbascoside content were selected. The π-ratio (low vs. high metabolite content) and *F*st were calculated using VCFtools with a sliding window of 20 kb and a step size of 10 kb. Putative selective sweep regions were defined as those falling within the top 5% of both the π-ratio and *F*st distributions, with thresholds of *F*st ≥ 0.36 and π-ratio ≥ 1.265.

### Determination of the verbascoside by high-performance liquid chromatography (HPLC)

A total of 1 g sample from each of 95 *C. nudiflora* accessions was extracted with 50 ml 70% methanol by ultrasonic treatment (40 kHz, 400 W) for 20 min and filtered through a 0.22-μm membrane before HPLC analysis. Verbascoside standard was prepared at 2.16 mg·ml^−1^ in 70% methanol as stock solution. HPLC was performed on an Agilent 1260 system with a TC-C18 column (4.6 × 250 mm, 5 μm) using 0.4% phosphoric acid (A) and acetonitrile (B) as mobile phases under a gradient (8%–16% B in 0–30 min, 16%–21% B in 30–45 min, 21%–24% B in 45–55 min, 24%–35% B in 55–65 min, up to 100% B at 70 min). Flow rate was 1 ml/min; injection volume, 10 μl; detection at 330 nm (Fig. S16).

Calibration curves were established by analyzing a series of standard solutions, which were prepared by diluting the stock solution at dilution factors of 0-, 2.5-, 5-, 10-, 50-, and 100-fold, respectively. Linear regression was performed with concentration as the abscissa and peak area as the ordinate. The limits of detection (LOD) and quantification (LOQ), precision, repeatability, stability, and recovery were evaluated to validate the HPLC method. LOD and LOQ were determined based on the signal-to-noise ratio (S/N). The stock solution was serially diluted until the S/N reached approximately three for LOD and 10 for LOQ, which defined the corresponding concentrations. Precision was assessed by performing six replicate injections of the stock solution on the HPLC system, while repeatability was confirmed by analyzing six independently prepared samples. Stability was tested by analyzing the sample solution at 0, 2, 4, 8, 12, and 24 h. Recovery was determined by spiking known amounts of standards into 0.5 g sample (Table S7).

### Widely targeted plant metabolomics

Freeze-dried mature leaves, stems, roots, and flowers were ground in a mixer mill (MM 400, Retsch) with a zirconia bead for 1.5 min at 30 Hz. A total of 100 mg powder was extracted overnight at 4°C with 1.2 ml of 70% methanol. After centrifugation (10 000 g, 10 min), the supernatant was purified using a CNWBOND Carbon-GCB SPE cartridge (250 mg, 3 ml; ANPEL) and filtered through a 0.22-μm membrane (SCAA-104; ANPEL) before UPLC-MS/MS analysis.

Metabolite profiling was performed on an UPLC-ESI-MS/MS system (Shim-pack UFLC SHIMADZU CBM30A; Applied Biosystems 6500 Q TRAP). Separation used a Waters ACQUITY UPLC HSS T3 C18 column (1.8 μm, 2.1 × 100 mm) with solvent A (water +0.04% acetic acid) and solvent B (acetonitrile +0.04% acetic acid). The gradient was: 0–10 min, 95% to 5% A; 10–11 min, 5% A; 11–11.1 min, return to 95% A; 11.1–14 min, equilibration at 95% A. Column temperature was 40°C; injection volume, 2 μl.

Mass spectrometry used a triple quadrupole-linear ion trap (API 6500 Q TRAP) with ESI Turbo Ion-Spray in positive and negative modes, controlled by Analyst 1.6.3. Parameters: source temperature 550°C; ion spray voltage +5500 V/−4500 V; gas I 50 psi; gas II 60 psi; curtain gas 30 psi; and collision gas high. Instrument tuning and calibration used polypropylene glycol solutions (10 and 100 μmol/L) in QQQ and LIT modes. MRM experiments were conducted with nitrogen at 5 psi; DP and CE were optimized per transition. MRM transitions were monitored according to metabolite retention times.

### Heterologous expression and protein purification

Total RNA from *C. nudiflora* leaves was extracted with TranZol™ (TransGen Biotech, China), and cDNA was synthesized using TransScript SuperMix. Target genes (Table S6) were amplified with TransStart FastPfu DNA Polymerase and cloned into pET28a(+) (*Bam*HI site) via seamless cloning. Verified constructs were transformed into *E. coli* BL21 (DE3). Cultures in LB + kanamycin (50 μg/ml) were grown at 37°C to OD_600_≈ 0.6, induced with 0.1 mM IPTG at 18°C for 18–24 h, and harvested (7800 rpm, 4°C, 3 min). Pellets were resuspended in lysis buffer (20 mM Tris–HCl, pH 7.4; 200 mM NaCl; 2% glycerol), sonicated on ice (15 min), and centrifuged (7800 rpm, 4°C, 20 min). The supernatant was purified on Ni-NTA resin, eluted with 30 and 300 mM imidazole, concentrated (Amicon Ultra-15, 30 K), and stored (−80°C) in buffer with 20% glycerol.

CYP450 genes were cloned into pESC-Leu (*Bam*HI site) by homologous recombination and transformed into *S. cerevisiae* WAT11. Transformants were selected on SD/-Leu plates and grown in SD/-Leu overnight at 30°C. Cells were resuspended (OD_600_ = 1.0) in SG/-Leu medium and induced for 24 h. Cells were disrupted in extraction buffer (50 mM Tris–HCl, pH 7.4; 1 mM EDTA; 0.6 M sorbitol) with glass beads (8 cycles, vortex 30 s/cool 30 s). After centrifugation (10 000 × g, 20 min), the supernatant was ultracentrifuged (160 000 × g, 90 min, 4°C). Microsomal pellets were resuspended in TEG buffer (50 mM Tris–HCl, pH 7.4; 1 mM EDTA; 20% glycerol).

### Enzymatic activity assays

GT assays (50 μl) contained 50 mM Na₂HPO₄–NaH₂PO₄ (pH 6.0), 0.1 mM substrate, 0.5 mM sugar donor, and 30 μg purified enzyme, incubated at 30°C for 3 h. AT assays (50 μl) contained 50 mM Tris–HCl (pH 6.0), 0.2 mM substrate, 0.5 mM acyl donor, and 30 μg purified enzyme, incubated at 30°C for 2 h. P450 assays (50 μl) contained 50 mM Tris–HCl (pH 6.0), 0.5 mM NADPH, 0.1 mM substrate, and 20 μl yeast microsomes, incubated at 30°C for 3 h. Reactions were stopped with 100 μl MeOH, centrifuged (15 000 rpm, 20 min), and supernatants analyzed. Controls used heat-denatured enzymes.

Samples were separated on a Zorbax SB-C18 column (4.6 ×250 mm, 5 μm) with a Zorbax Extend-C18 guard column at 35°C; flow rate: 1 ml/min; mobile phase: water +0.1% formic acid (A) and acetonitrile (B); and gradient: 0–4 min, 5% B; 4–24 min, 5%–30% B; 24–26 min, 30%–100% B; 26–26.1 min, 100%–5% B; 26.1–29 min, 5% B. Filtered samples (0.22 μm) were separated on a Waters T3 column (2.1 × 100 mm, 1.8 μm) at 50°C; flow rate: 0.3 ml/min; mobile phase: water +0.1% formic acid (A) and methanol (B); and gradient: 0–4 min, 20%–35% B; 4–7 min, 35%–40% B; 7–11.5 min, 40%–100% B; 11.5–13 min, 100% B; 13–13.1 min, 100%–20% B; 13.1–16 min, 20% B. MS was run in negative ion mode (spray voltage −3.5 kV; capillary, 350°C; sheath gas, 45 arb; aux gas, 10 arb; probe heater, 400°C; S-lens RF, 60 V; resolution, 70 000 for full MS and 17 500 for MS/MS; scan range m/z, 100–1200; NCE, 35 eV). Data were processed with Xcalibur™ 4.1 (Thermo Fisher Scientific).

## Supplementary Material

Web_Material_uhag203

## Data Availability

Data supporting the findings of this study are available in the article, supplementary materials, or public database. Source data are provided with this paper.
