# Supplementary material for: Deciphering genetic basis of phenylethanoid glycoside production through integrative multiomics analysis in Callicarpa nudiflora
Source: Hortic Res. 2026 Jun 10;13(10):uhag203. doi: 10.1093/hr/uhag203 (PMC13641927; doi:10.1093/hr/uhag203)
Supplement: Supplementary file 1 [file web_material_uhag203.zip › Supplementary Information-20260417.docx]

**Supplemental information**

**Deciphering genetic basis of phenylethanoid glycoside production through integrative multi-omics analysis in *Callicarpa nudiflora***

Fulai Yu^1,5*^, Mingju Yao^2,5^, Mei Huang^1,5^, Xunmeng Feng^3^, Lanlan Zang^3^, Donghua Hu^3^**,** Lanping Guo^4^, Shichao Sun^3,*^, Xue Qiao^2,*^, Li Wang^3,*^

**Supplemental Tables:**

**Table S1** Summary of assembly and annotations of the *C. nudiflora* and *C. americana* genomes.

**Table S2** Functional enrichment of species-specific gene families in the *C. nudiflora* genome.

**Table S3** Functional enrichment of contracted gene families in the *C. nudiflora* genome.

**Table S4** Sequencing depth and mapping rate of resequenced samples.

**Table S5** Accumulation patterns of active compounds in different tissues of *C. nudiflora*.

**Table S6** Primers for functional gene cloning in this study.

**Table S7** Method parameters and the calibration curves by HPLC.

**Supplemental Figures:**

**Figure S1** Putative biosynthetic pathway of PhGs based on minor modification of Figure 2 in Zhai et al., 2021.

**Figure S2** Somatic chromosome squash from a root tip cell of *C. nudiflor.* exhibits 2n = 34. Bar = 10 μm.

**Figure S3** Genome size and heterozygosity estimation based on K-mer analysis.

**Figure S4** Hi-C contact map of 17 chromosomes in C. nudiflora.

**Figure S5** BUSCO assessment of the genome assembly and annotation in C. nudiflora.

**Figure S6** Venn diagram illustrating the number of shared and species-specific gene families in C. nudiflora.

**Figure S7** Functional characterization of Cn06G0864 and Cn06G0865 using compound **1** as the substrate.

**Figure S8** Functional characterization of Cn05G1944 and Cn05G1945 using compound **2** as the substrate.

**Figure S9** Functional characterization of Cn02G0899 using compound **3** as the substrate.

**Figure S10** Functional Characterization of Cn05G2248 and Cn16G0232 using compound **4** as the substrate.

**Figure S11** Comparative synteny analysis of verbascoside biosynthetic genes across four Lamiales species.

**Figure S12** Expression profiling of candidate genes involved in the verbascoside biosynthesis pathway in C. nudiflora.

**Figure S13** Comparison of heterozygosity among C. nudiflora subpopulations.

**Figure S14** Catalytic activity of the two haplotypes of Cn02G0899 protein on the substrate osmanthuside A.

**Figure S15** 49 plant species with the highest content of verbascoside.

**Figure S16** HPLC chromatograms of the reference substance verbascoside (**A**) and of the 70% methanol ultrasonic extraction of leaves of one *C. nudiflora* sample(**B**).

**Table S1** Summary of assembly and annotations of the *C. nudiflora* and *C. americana* genomes.

**Table S2** Functional enrichment of species-specific gene families in the *C. nudiflora* genome.

**Table S3** Functional enrichment of contracted gene families in the *C. nudiflora* genome.

**Table S4** Sequencing depth and mapping rate of resequenced samples.

**Table S5** Accumulation patterns of active compounds in different tissues of *C. nudiflora*.

**Table S6** Primers for functional gene cloning in this study.

**Table S7** Method parameters and the calibration curves by HPLC.

**Figure S1** Putative biosynthetic pathway of PhGs based on minor modification of Figure 2 in Zhai et al., 2021. (1. Phenylalanine pathway; 2. Tyrosine pathway)

**Zhai, J.C.X.S., Zemin; LI,Chun; Lv,Bo** (2021). Current advances in biosynthesis of acteoside. China Biotechnology **41**:94-104. https://doi.org/10.13523/j.cb.2102012.

**Figure S2** Somatic chromosome squash from a root tip cell of *C. nudiflora* exhibits 2n = 34. Bar = 10 μm. Green fluorescence signal indicates 45S rDNA; red fluorescence signal represents 5S rDNA.

**Figure S3** Genome size and heterozygosity estimation based on K-mer analysis.

**Figure S4** Hi-C contact map of 17 chromosomes in C. nudiflora.

**Figure S5** BUSCO assessment of the genome assembly and annotation in C. nudiflora.

**Figure S6** Venn diagram illustrating the number of shared and species-specific gene families in C. nudiflora. (Cnud), C. americana (Came), Olea europaea (Oeur), Thymus quinquecostatus (Tqui), Salvia bowleyana (Sbow), Salvia hispanica (Shis), Scutellaria baicalensis (Sbai), Tectona grandis (Tgra), and two outgroups (Vitis vinifera (Vvin) and Ilex asprella (Iasp)).

**Figure S7** Functional characterization of Cn06G0864 and Cn06G0865 using compound **1** as the substrate. (**A**) Reaction catalyzed by Cn06G0864 and Cn06G0865. (**B**) HPLC/UV chromatograms of the reaction mixtures (275 nm). (**C**) MS and MS/MS analysis of the product (**2**) in the reaction mixture.

**Figure S8** Functional characterization of Cn05G1944 and Cn05G1945 using compound **2** as the substrate. (**A**) Reaction catalyzed by Cn05G1944 and Cn05G1945. (**B**) HPLC/UV chromatograms of the reaction mixtures (275 nm). (**C**) MS and MS/MS analysis of the product (**7**) in the reaction mixture.

**Figure S9** Functional characterization of Cn02G0899 using compound **3** as the substrate. (**A**) Reactions catalyzed by Cn02G0899. (**B**) HPLC/UV chromatograms of the reaction mixtures (275 nm). (**C**) MS and MS/MS analysis of the product (**4**) in the reaction mixture.

**Figure S10** Functional Characterization of Cn05G2248 and Cn16G0232 using compound **4** as the substrate. (**A**) Reactions catalyzed by Cn05G2248 and Cn16G0232. (**B**) MS and MS/MS analysis of the products in the reaction mixture.

**Figure S11** Comparative synteny analysis of verbascoside biosynthetic genes across four Lamiales species. (A-D) Microsynteny of genomic regions harboring verbascoside biosynthetic genes. Gray ribbons represent conserved syntenic blocks between *C. nudiflora* (Cnud), *C. americana* (Came), *Scutellaria baicalensis* (Sbai), and *Salvia bowleyana* (Sbow). Red ribbons highlight the specific positions and syntenic relationships of the target biosynthetic genes, including *Cn06G0864/65* (**A**), *Cn05G1944/45* (**B**), *Cn02G0899* (**C**), *Cn05G2248*, and *Cn16G0232* (**D**). The conserved collinearity across these species suggests that the functional redundancy of these enzymes in C. nudiflora primarily originated from ancient WGD or segmental duplication events. Chromosome IDs and genomic coordinates (Mb) are indicated for each respective segment.

**Figure S12** Expression profiling of candidate genes involved in the verbascoside biosynthesis pathway in C. nudiflora.

**Figure S13** Comparison of heterozygosity among C. nudiflora subpopulations.

**Figure S14** Catalytic activity of the two haplotypes of Cn02G0899 protein on the substrate osmanthuside A.

Statistics of 49 known plant species with the highest content of verbascoside(mg/g, dw)

|  | 49种含有毛蕊花糖苷植物的最高含量报道(mg/g, dw) |
| --- | --- |
| Sideritis trojana | 0.02 |
| Scutellaria galericulata | 0.028 |
| Callicarpa peii | 0.065 |
| Hypericum sampsonii | 0.24 |
| Osmanthus fragrans | 0.62 |
| Pedicularis dichotoma | 1.59 |
| Plantago ovata | 1.7 |
| Scrophularia ningpoensis | 1.86 |
| Verbascum phoeniceum | 2 |
| Lagopsis supina | 2.48 |
| Plantago major | 3.61 |
| Pedicularis decora | 5.15 |
| Plantago depressa | 6.42 |
| Phlomoides umbrosa | 6.82 |
| Cistanche lanzhouensis | 6.97 |
| Siphonostegia chinensis | 7.04 |
| Callicarpa kochiana | 7.52 |
| Verbascum densiflorum | 8 |
| Castilleja tenuiflora | 9.23 |
| Lindernia ruellioides | 9.44 |
| Pedicularis flava | 9.7 |
| Ligustrum lucidum | 10.52 |
| Phlomoides younghushandii | 10.59 |
| Cistanche salsa | 10.9 |
| Pogostemon auricularius | 11.27 |
| Plantago asiatica | 11.4 |
| Phlomoides rotata | 11.73 |
| Verbascum phlomoides | 13 |
| Lagotis alutacea | 13.95 |
| Verbascum xanthophoeniceum | 16 |
| Matsumurella chinense | 17.6 |
| Olea europaea | 29 |
| Pedicularis muscicola | 29 |
| Verbascum nigrum | 30 |
| Cistanche deserticola | 31.53 |
| Callicarpa kwangtungensis | 31.9 |
| Callicarpa macrophylla | 33.03 |
| Pedicularis Kansuensis | 41.6 |
| Buddleja officinalis | 42.89 |
| Callicarpa dolichophylla | 43.28 |
| Verbena officinalis | 50 |
| Lantana camara | 52 |
| Callicarpa parvifolia | 53.21 |
| Rehmannia glutinosa | 59.68 |
| Cistanche tubulosa | 65 |
| Lancea tibetica | 71.1 |
| Cistanche sinensis | 88.12 |
| Lippia citriodora | 118 |
| Callicarpa nudiflora | 136.39 |

**Figure S15** 49 plant species with the highest content of verbascoside.

**B**

**A**

**Figure S16** HPLC chromatograms of the reference substance verbascoside (**A**) and of the 70% methanol ultrasonic extraction of leaves of one *C. nudiflora* sample(**B**).
