# Supplementary material for: Deciphering genetic basis of phenylethanoid glycoside production through integrative multiomics analysis in Callicarpa nudiflora
Source: Hortic Res. 2026 Jun 10;13(10):uhag203. doi: 10.1093/hr/uhag203 (PMC13641927; doi:10.1093/hr/uhag203)
Supplement: Supplementary file 1 [file web_material_uhag203.zip › Table S8(for comment 6)-20260417.pdf]

Table S8 Summary of the Genetic Transformation System for Woody Plants

No.	Species	Genus, Family	Transformation conditions	Transformation efficiencies	Transformation time	Difficulty	References	Year	Remarks
1	*Lycium ruthenicum*	Lycium, Solanaceae	Method: Agrobacterium-mediated transformation<br>Explants: Leaves from tissue-cultured seedlings<br>Agrobacterium concentration: OD600= 0.2<br>Infection time: 10 minutes<br>Acetosyringone (AS): 200 µmol/L<br>Co-cultivation period: 2 days<br>Co-cultivation medium: MS + 0.5 mg/L 6-BA + 0.5 mg/L NAA<br>Selective antibiotic: 40 mg/L Hygromycin<br>Bacteriostatic antibiotic: 200 mg/L Carbenicillin	Mean Transformation Efficiency: 1.18%-5.33%；<br>Gene Editing Efficiency: 95.45%	Overall Timeline: 2 months	1.Complex genomes<br>2. Slow growth cycles<br>3. Difficulty in transformation<br>4. Lack of genomic information<br>5. Unknown genome sequence<br>6. Lack of efficient regeneration systems<br>7. Lack of efficient genetic transformation systems	Wang W , Jiangmiao L , Hai W , et al. A highly efficient regeneration, genetic transformation system and induction of targeted mutations using CRISPR/Cas9 in Lycium ruthenicum.[J]. Plant methods, 2021,17(1): 71-71.DOI: 10.1186/S13007-021-00774-X.	2021	Bush
2	*Hevea brasiliensis*	Hevea, Euphorbiaceae	Explant: Cotyledonary somatic embryos<br>Agrobacterium strain: EHA 105<br>Vector: pCAMBIA 2301<br>Bacterial density: OD600 = 0.45<br>Infection method: 8 min incubation → 50 s sonication → 10 min incubation<br>Sonication: 50 seconds, 40 kHz<br>Preculture: Not required<br>Acetosyringone: 100 µmol/L<br>Co-cultivation temperature: 22°C<br>Co-cultivation duration: 84 hours (3.5 days), dark<br>Selection antibiotic: Kanamycin 100 mg/L<br>Bacteriostatic antibiotic: Timentin 500 mg/L	The GUS-positive embryo rate was approximately 60%, with an average of 88.67 GUS spots per embryo.	Total duration: From infection to obtaining resistant embryos, approximately 2 months；<br>Co-cultivation duration: 84 hours (3.5 days) at 22°C in darkness；<br>Recovery: 2 days without antibiotics；<br>Resistant embryo induction: Globular embryos appear after 16 days；<br>Resistant embryo maturation: Torpedo-shaped resistant embryos obtained after 40 days.	1. Low bacterial infection rate, affecting transformation success<br>2. Inability of bacterium to reach inner cells of plant tissues, limiting transformation efficiency<br>3. Risk of chimeras, in which transgene integration occurs only in some cells, resulting in mixed tissues<br>4. Mechanisms of escape formation and optimal antibiotic dose requiring further investigation<br>5. Recurring growth of bacterium in newly developed resistant cells, difficult to eliminate<br>6. Low transgenic seedling regeneration from embryogenic calli, leading to unsatisfactory transformation efficiency	Jinu U ,Tiandai H , Shichao X , et al.Optimization of the Transformation Protocol for Increased Efficiency of Genetic Transformation in Hevea brasiliensis[J].Plants,2022,11(8): 1067-1067.DOI:10.3390/PLANTS11081067.	2022	Woody Plant
3	*Manihot esculenta* Crantz	Manihot, Euphorbiaceae	1.Agrobacterium Strain & Vector<br>Agrobacterium strain: LBA4404<br>Vector Backbone: pCAMBIA2300<br>Markers: nptII (selectable, paromomycin resistance); GFP or DsRed (visual)<br>2.Target Tissue Preparation<br>Explant: Young leaf lobes<br>OES induction: MS2 + 50 µM picloram + 2 µM $CuSO_4$<br>FEC induction: GD2 + 50 µM picloram<br>3.Inoculation & Co-culture<br>OD600: 0.5<br>Inoculation time: 30min<br>Co-culture time: 2 days<br>Temperature: 22°C	Transformation Efficiency in : 20–30 independent transgenic events per cm^3 SCV	Co-culture to plant recovery: 3–3.5 months,4 months for other cultivars;<br>Explant to FEC preparation: 3 months,3–4 months for other cultivars;<br>Transformation to greenhouse: 4–5 months,5–6 months for other cultivars.	1. Strong genotype dependence<br>2. Complex tissue culture system<br>3. High inter - cultivar variability<br>4. Explant contamination<br>5. Failure of shoot emergence<br>6. Poor growth of mother plants	Rosana S ,Tira J ,Danielle S , et al.Agrobacterium-mediated Genetic Transformation of Cassava.[J].Current protocols, 2022,2(12):e620-e620.DOI:10.1002/CPZ1.620.	2022	Woody Plant
4	*Poplar*	Populus, Salicaceae	Method: Particle bombardment<br>Target tissue: Sterile leaves<br>Selection marker: aadA<br>Integration sit: Plastid genome<br>Foreign gene: GFP<br>Homoplasmy: Obtained after multiple selection rounds	1.Tobacco<br>Transformation efficiency: High; aadA gene increases transformation frequency 100-fold;<br>Protein expression level: Up to 72% TSP;<br>Homoplasmy: Achieved after 2–3 selection rounds.<br>2.Rice<br>Efficiency: Still low; remains a "proof of concept"<br>3.Wheat Efficiency: Unconfirmed; pending further experimentation<br>4.Maize<br>Transformation status: Never successfully transformed;<br>Transient expression: Green fluorescent points observed in scutellum;<br>5.Poplar<br>Transformation status: Successfully transformed;<br>Foreign protein expression: Expressed in chloroplasts	Vector construction: Weeks to months;<br>Bombardment & initial culture: 1–2 days;<br>1st selection round: 4–6 weeks;<br>2nd selection round: 4–6 weeks;<br>3rd selection round: 4–6 weeks;<br>Regeneration & rooting: 4–8 weeks;<br>Greenhouse transfer: 1–2 weeks.	1.Recalcitrance of monocots<br>2.Low transformation efficiency<br>3.Difficulty in obtaining homoplasmy<br>4.Low expression in non-green tissues	Quintín Rascón-Cruz,Carmen Daniela González-Barriga, Iglesias-Figueroa B F ,et al.Plastid transformation: advances and challenges for its implementation in agricultural crops[J].Electronic Journal of Biotechnology, 2021 (1).DOI:10.1016/j.ejbt.2021.03.005.	2021	Arbor

5	*Vitis spp.*	Vitis, Vitaceae	Method:<br>Biolistic bombardment,Agrobacterium-mediated transformation<br>Acceptor Materials:<br>Embryogenic callus, Proembryonic masses, Somatic embryos, Shoot apical meristems, Suspension cultures<br>Agrobacterium Strain & Concentration:<br>Common strains: EHA105,LBA4404,GV3101,C58<br>Efficient strain: EHA105 yields the highest transformation efficiency Bacterial density: OD600 = 0.06–0.2<br>Infection time: 5–30 minutes<br>Co-cultivation Conditions<br>Medium: Both solid and liquid; liquid followed by solid improves efficiency<br>Co-cultivation duration: 2–5 days<br>Temperature: Suitable temperature (22–25°C), typically in the dark<br>Filter paper: Two layers of sterile filter paper during co-cultivation<br>Selectable Markers & Selection Agents<br>nptII,Kanamycin,10–150 mg/L<br>hptII,Hygromycin,Optimized per cultivar<br>Bar,Phosphinothricin,Optimized per cultivar<br>egfp / gus,Visual screening,Used together with antibiotics<br>VvMybA1,Anthocyanin visual marker,No antibiotic selection needed<br>Antibiotics for Agrobacterium Suppression<br>Cefotaxime: 200–500 mg/L<br>Carbenicillin: 200–500 mg/L<br>Culture Media<br>EC induction: MS,NN,NB,DM,GS1CA,X6	1.Efficiency of Different Transformation Methods<br>Agrobacterium-mediated: Relatively highest; most effective method<br>Biolistic bombardment: Lower; transgenic plants obtained but efficiency variable<br>A.rhizogenes-mediated: Only transgenic roots obtained; plant regeneration difficult<br>Protoplast transformation: Mainly for transient expression; stable transformation difficult<br>2.Key Factors Affecting Transformation Efficiency<br>①Genotype<br>Thompson Seedless: Relatively high; most commonly used<br>Chardonnay: Relatively high<br>Sugraone,Crimson Seedless: Moderate<br>Other cultivars: Low; transformation difficult<br>②Explant Type<br>Somatic embryos (SE): Highest<br>Proembryonic masses (PEM): High<br>Embryogenic callus (EC): Moderate<br>Shoot apical meristems, Leaves: Lower<br>③Agrobacterium Strain & Concentration<br>EHA105: Highest<br>LBA4404,GV3101: Moderate<br>C58(pMP90): Lower	Pre-culture duration: 7 days;<br>Infection time: 5–30 min;<br>Co-cultivation time: 2–5 days;<br>Selection rounds: Multiple rounds;<br>Total transformation timeline: typically several months.	1.Recalcitrance of most cultivars<br>2.Organogenesis leads to chimeras<br>3.Low efficiency with stage dependence<br>4.Necrosis from high bacterial density<br>5.Browning after infection<br>6.False results from improper concentration<br>7.Failure of regeneration in some cultivars<br>8.Residual marker genes	Zhang X M , Yi-Fei W U , Zhi L I ,et al.Advancements in plant regeneration and genetic transformation of grapevine (Vitis spp.)[J].Journal of Integrative Agriculture, 2021, 20(6):1407-1434.DOI:10.1016/S2095-3119(20)63586-9.	2021	Woody Plant
6	*Passiflora edulis Sims*	Passiflora, Passifloraceae	Method:<br>Agrobacterium-mediated transformation,Biolistic bombardment,In planta transformation, Nanoparticle-mediated delivery<br>Acceptor Materials and Explants<br>Stem segment,Leaf,Anther, Hypocotyl<br>Agrobacterium Strains<br>EHA105,<br>LBA4404<br>GV3111SE<br>Infection & Co-cultivation Conditions<br>Bacterial density: OD600 = 0.5<br>Infection time: 20–30 minutes<br>Acetosyringone (AS): 2 × 10-4 mol/L(200 µmol/L)<br>Co-cultivation time: 3 days<br>Light condition: Dark culture promotes shoot formation<br>Sonication-assisted: 30s sonication significantly improves efficiency	Highest efficiency: 57.89%;<br>In planta efficiency: 29%;<br>Conventional Agrobacterium efficiency: 0.11%–11.67%.	In planta transformation total: 5–8 weeks;<br>KPF4 hybrid regeneration total: 4 weeks;<br>Dark culture duration: 15 days;<br>Natural light culture duration: 5–8 weeks.	1.Low efficiency of conventional Agrobacterium<br>2.Instability and poor reproducibility<br>3.Chimeras from organogenesis<br>4.Culture period in indirect pathways<br>5.Significant variation among cultivars<br>6.Unstable expression in some plants	Junle N,Zheng T,Cuifeng Y, et al.Reserach Progress on in Regemeration and Genetic Transformation in Passion Fruit [J].China Fruit & Vegetable,2026,46(01):54-63.DOI:10.19590/j.cnki.1008-1038.2026.01.009.	2026	Woody Vine
7	*Rhododendro* spp.	Rhododendron, Ericaceae	Method: Agrobacterium-mediated<br>Acceptor material: Leaf (highest efficiency), stem segment, callus<br>Pre-culture duration: 2–5 days<br>Agrobacterium strain: GV3101, EHA105, LBA4404, AGL1<br>Bacterial density: OD600 = 0.4–1.0<br>Infection time: 15–40 min<br>Co-cultivation time: 2–3 days<br>Antibiotics for suppression: Timentin, Cefotaxime, Carbenicillin<br>Selective antibiotics: Kanamycin, Hygromycin<br>Acetosyringone (AS): 20 mg/L (co-culture); 50 mmol/L (infection)	Highest efficiency: 70% (R. yunnanense leaf);<br>Highest resistant callus frequency: 37% (R. simsii, with AS).	Pre-culture duration: 2–5 days;<br>Infection time: 15–40 min;<br>Co-cultivation time: 2–3 days;<br>Antibiotic & selection culture: Several weeks;<br>Plant regeneration: Weeks to months;<br>Total timeline: typically months to half a year.	1.Browning due to high phenolic content<br>2.Difficulty in eliminating bacteria from callus<br>3.High inter-cultivar variation<br>4.Impact of treatment duration on efficiency<br>5.High concentration inhibiting growth; low concentration causing false positives<br>6.Lack of studies on somatic embryogenesis	Yuan Z,Bei H,Jun T, et al.Regeneration and Genetic Transformation System of Rhododendron[J/OL].Molecular Plant Breeding,1-15[2026-03-29].https://link.cnki.net/urlid/46.1068.S.20251107.1819.002.	2025	Bush
8	*Eucalyptus urophylla × Eucalyptus grandis*	Eucalyptus, Myrtaceae	Explant: Stem segment (4–8 mm)<br>Pre-culture time: 6 days<br>Agrobacterium strain: GV3102<br>Bacterial density: OD600 = 0.3<br>Infection solution pH: 5.8<br>Infection time: 2.0 h<br>Co-culture time: 2 days<br>Antibiotic: Cefotaxime 200 mg/L<br>Selectable marker: mCherry (red fluorescence)<br>Bud differentiation rate: 64.36%<br>Fluorescence positive rate: 54.7%<br>Overall efficiency: 42%	Optimal bud differentiation rate: 64.36%;<br>Optimal fluorescence positive rate: 54.7%;<br>Overall transformation efficiency: 42%.	Pre-culture: 6 days;<br>Infection: 2.0 h;<br>Co-cultivation: 2 days;<br>Antibiotic selection culture: 30 days;<br>Elongation culture: Until 2–3 cm;<br>Rooting culture: Until 5 cm;<br>Total timeline: approximately 1.5–2 months (from explant to rooted plantlets).	1.High false positives and low efficiency in antibiotic screening<br>2.Chimeras from non-singl-cell explants<br>3.Key parameters not systematically optimized<br>4.High density or long infection causes necrosis<br>5.Impact of treatment duration on efficiency<br>6.No previous application of mCherry in Eucalyptus	Zechen W ,Limei L ,Lejun O .Efficient genetic transformation method for Eucalyptus genome editing.[J].PloS one,2021,16(5):e0252011-e0252011.DOI:10.1371/JOURNAL.PONE.0252011.	2021	Arbor

9	*Morus* L.	Morus, Moraceae	Method: Sonication + vacuum infiltration<br>Best genotype: ‘Taiguo’<br>Agrobacterium strain: LBA4404<br>Bacterial density: OD600 = 0.5<br>Acetosyringone: 150 µM<br>Surfactant: 0.02% Silwet L-77<br>Sonication time: 20 seconds<br>Vacuum pressure: 0.07 MPa<br>Vacuum infiltration time: 20 minutes<br>Co-cultivation time: 4 days<br>Detection time: 7 days<br>Reporter gene: GFP	Best genotype: ‘Taiguo’;<br>Best Agrobacterium strain: LBA4404;<br>Optimal OD600: 0.5;<br>Optimal AS concentration: 150 µM;<br>Optimal sonication duration: 20 s;<br>Optimal detection time: 7 days;<br>Efficiency improvement: Sonication significantly enhances efficiency;<br>Functional validation efficiency: Highly significant anthocyanin accumulation achieved.	Sonication: 20 s;<br>Vacuum infiltration: 20 min;<br>Co-cultivation: 4 days;<br>Earliest detection: 4 days;<br>Peak expression: 7 days;<br>Expression duration: 10–15 days;<br>Functional validation observation: 5–7 days;<br>Total experimental period: Approximately 1–2 weeks.	1. Syringe infiltration limited by leaf structure<br>2. Low efficiency of traditional methods<br>3. Large variation among genotypes<br>4. Necrosis from sonication<br>5. Cell death from high density<br>6. Applicability limited to few genotypes	Mo R ,Zhang N ,Qiu C , et al.Refinement and Enhancement of Agrobacterium-Mediated Transient Transformation for Functional Gene Examination in Mulberry (Morus L.)[J].Genes,2024,15(10):1277-1277.DOI:10.3390/GENES15101277.	2024	Woody Plant
10	*1.Pinus taeda,Pinus radiata,Pinus tabuliformis,Pinus strobus*<br>*2.Picea glauca,Picea mariana,Picea abies*<br>*3.Larix decidua,Larix kaempferi,Larix olgensis*<br>*4.Abies nordmanniana,Abies koreana*<br>*5.Cunninghamia lanceolata*<br>*6.Cryptomeria japonica*<br>*7.Taxus spp.*	Pinus, Picea, Larix, Abies, Cunninghamia, Cryptomeria, Taxus, Pinaceae, Cupressaceae, Taxaceae	Main method: Agrobacterium-mediated<br>Common strains: EHA105, GV3101, LBA4404<br>Bacterial density: OD600= 0.3–0.8<br>Infection time: Species-dependent<br>Promoter: CaMV 35S；PcUbi,Cula11<br>Antibiotics for suppression: Cefotaxime,Timentin,Carbenicillin<br>Selectable markers: nptII,hpt,bar<br>Reporter genes: GUS, GFP, LUC<br>Best explants: Embryogenic tissues<br>Optimizing additives: Acetosyringone, sonication, coniferyl alcohol	Highest transient efficiency: 70.1% (P. tabuliformis callus);<br>Highest stable efficiency: 1–2 events per 100 explants (hybrid larch).	Co-cultivation time: 2–5 days;<br>Antibiotic & selection culture: Weeks to months;<br>Somatic embryogenesis: Weeks to months;<br>Plant regeneration: Weeks;<br>Total timeline: Several months or longer.	1.Extremely low efficiency<br>2.Poor tissue culture with difficult regeneration<br>3.Large variation among genotypes<br>4.Low activity of the 35S promoter<br>5.Inhibit enzymes and oxidative stress<br>6.Hinders to DNA entry<br>7.Cytotoxicity<br>8.Overgrowth and necrosis	Zhao H ,Zhang J ,Zhao J , et al.Genetic transformation in conifers: current status and future prospects.[J].Forestry research,2024,4e010.DOI:10.48130/FORRES-0024-0007.	2024	Woody Plant
11	*Eucalyptus grandis , Eucalyptus urophylla , E. urophylla × E. grandis , E. grandis × E. urophylla , Eucalyptus globulus , Eucalyptus nitens , Eucalyptus camaldulensis , Eucalyptus tereticornis , Eucalyptus saligna , Eucalyptus citriodora , Eucalyptus dunnii , Eucalyptus cloeziana , Eucalyptus polybractea , Eucalyptus benthamii , Eucalyptus pellita , Eucalyptus bosistoana , E. maidenii × E. saligna , E. grandis × E. camaldulensis , E. urophylla × E. tereticornis , E. grandis × E.*	Eucalyptus , Myrtaceae	Main method: Agrobacterium-mediated<br>Common strains: EHA105, GV3101, AGL1<br>Common explants: Hypocotyls, stem segments, leaves, cotyledons<br>Pre-culture time: 2–7 days<br>Bacterial density: OD600= 0.3–0.8<br>Infection time: 30 min – 2 h<br>Co-culture time: 2–5 days<br>Antibiotics for suppression: Cefotaxime, Timentin 100–200 mg/L<br>Selective antibiotics: Kanamycin 15–75 mg/L; Hygromycin 5–15 mg/L<br>Additives: Acetosyringone 100–150 µM; sonication 30 s	Highest efficiency: 42% (stem segment, fluorescence);<br>Second highest: 40% (hypocotyl, fluorescence);<br>Conventional Agrobacterium efficiency: 1.5%–24%;<br>A.rhizogenes efficiency: 20.2%;<br>Sonication improvement: 93% increase.	Pre-culture: 6–7 days;<br>Infection: 30 min – 2 h;<br>Co-culture: 2–5 days;<br>Callus induction: 15 days;<br>Shoot induction and selection: Weeks to months (up to 180 days);<br>Shoot elongation, Rooting culture: Weeks;<br>Total timeline: Approximately 1.5–6 months.	1.Low efficiency<br>2.Large variation among genotypes<br>3.Browning due to phenolic compounds<br>4.False results from improper concentration<br>5.Necrosis from overgrowth<br>6.Lack of universal protocol<br>7.Low frequency of abnormal embryos	Wang Y ,Wu Z ,Li X , et al.Regeneration and Genetic Transformation in Eucalyptus Species, Current Research and Future Perspectives[J].Plants,2024,13(20):2843-2843.DOI:10.3390/PLANTS13202843.	2024	Arbor

12	*Eucalyptus urophylla,Eucalyptus grandis,Eucalyptus camaldulensis,Eucalyptus robusta,Eucalyptus pellita,Eucalyptus tereticornis,Eucalyptus dunnii,Eucalyptus globulus,Eucalyptus benthamii,Eucalyptus citriodora,E. exserta,Eucalyptus variegata*	Eucalyptus, Myrtaceae	Method: Agrobacterium-mediated<br>Agrobacterium strain: GV3101 (pMP90)<br>Vector: pCAMBIA2300: 35S: DsRed2<br>Reporter gene: DsRed2 (red fluorescence)<br>Selectable marker: nptII (kanamycin resistance)<br>Explant: Hypocotyl (best), internode<br>Pre-culture time: 3 days<br>Infection time: 30 min<br>Co-culture time: 4 days<br>Co-culture light: Dark<br>Selection concentration: Kanamycin 30 mg/L<br>Regeneration media: SIM1,SIM3<br>Best species: E. robusta (RO1),E. camaldulen (CA1)	Highest transient efficiency: 61.48% (E. robusta hypocotyl);<br>Highest stable efficiency: 3.9% (E. robusta).	Pre-culture: 3 days;<br>Infection: 30 min;<br>Co-cultivation: 4 days;<br>Selection & regeneration: 8 weeks;<br>Shoot elongation: 4 weeks;<br>Rooting: 2 weeks;<br>Total: Approximately 3–4 months.	1. Low and unstable efficiency<br>2. Large variation among species<br>3. Difficulty in controlling kanamycin concentration<br>4. Lower efficiency of internodes compared to hypocotyls<br>5. Lack of universal protocol	Zhou T ,Lin Y ,Lin Y , et al.Regeneration and Agrobacterium-mediated genetic transformation of twelve Eucalyptus species.[J].Forestry research,2022,215.DOI:10.48130/FR-2022-0015.	2022	Arbor
13	*Casuarina equisetifolia*	Casuarina, Casuarinaceae	Method: A. tumefaciens,A. rhizogenes<br>Strain: GV3101（A. tumefaciens）,K599（A. rhizogenes）<br>Vector: pCAMBIA1301（A. tumefaciens）,pCAMBIA1300-35S: GFP（A. rhizogenes）<br>Reporter: GUS（A. tumefaciens）,GFP（A. rhizogenes）<br>Selection marker: Hygromycin (5 mg/L)（A. tumefaciens）<br>Suppression antibiotic: Carbenicillin (250 mg/L)（A. tumefaciens）<br>Acetosyringone: 20 mg/L（A. tumefaciens）,100 μM（A. rhizogenes）<br>Infection time: 2 h（A. tumefaciens）,2–4 h（A. rhizogenes）<br>Co-cultivation time: 2 days（A. tumefaciens）<br>Efficiency: Transgenic plants obtained（A. tumefaciens）,95.9%（A. rhizogenes）<br>Total timeline: 4.5 months（A. tumefaciens）,45 days（A. rhizogenes）	A.rhizogenes hairy root induction efficiency: 28.3% (K599 strain);<br>A. rhizogenes transformation efficiency: 95.9% (positive hairy roots);<br>A. tumefaciens stable transformation efficiency: confirmed by GUS staining and PCR.	Method: A. tumefaciens;<br>Callus induction: 10–15 days;<br>Infection time: 2 h;<br>Co-cultivation: 2 days;<br>Selection and regeneration: 2–4weeks + 1–2 months;<br>Rooting: 1–2 weeks;<br>Total: 4.5 months.	1. Lack of efficient regeneration systems<br>2. Callus prone to browning<br>3. Difficulty in determining hygromycin concentration<br>4. Overgrowth from prolonged co-cultivation<br>5. Bud browning caused by cefotaxime<br>6. Low efficiency without As<br>7.Zero efficiency with the C58C1 strain<br>8.Protocols for other species not applicable	Huimin R ,Yan X ,Xiaohong Z , et al.Optimization of Tissue Culturing and Genetic Transformation Protocol for Casuarina equisetifolia [J].Frontiers in Plant Science,2022,12784566-784566.DOI:10.3389/FPLS.2021.784566.	2022	Woody Plant
14	*Citrus*	Citrus， Rutaceae	Main method: Agrobacterium-mediated<br>Common strains: EHA105, EHA101, LBA4404, C58<br>Best explant: Epicotyls, stem segments<br>Bacterial density: 4 × 107 – 5 × 108cfu/mL<br>Infection time: 5–20 min<br>Co-cultivation time: 2–3 days<br>Co-cultivation temperature: 19–28°C<br>Co-cultivation light: 2–4 weeks dark<br>Key additives: Acetosyringone, 2,4-D, BAP<br>Selectable markers: nptII (kanamycin), hpt (hygromycin), bar (Basta), PMI (mannose)<br>Reporter genes: GUS, GFP, DsRed2<br>Regeneration technique: Micrografting<br>Efficiency range: 0–45% (varies by genotype)	Agrobacterium efficiency range: 0–45%;<br>Best species: Poncirus, Citrus hybrids;<br>Most recalcitrant: Mandarin;<br>Best explant: Epicotyls, stem segments;<br>Best strains: EHA105, EHA101, A281;<br>PMI/mannose efficiency: 1.9% (biolistic);<br>Cre/loxP marker removal efficiency: 21.4%;<br>CRISPR editing efficiency: 23–75%;<br>Pollen magnetofection efficiency: 5% (5 plants/100 flowers).	Agrobacterium infection time: 5–20 min;<br>Co-cultivation time: 2–3 days;<br>Dark culture duration: 2–4 weeks;<br>Selection and regeneration: Weeks to months;<br>Greenhouse after micrografting: Weeks;<br>Mature tissue to flowering: 14 months;<br>Juvenile tissue to flowering: >10 years;<br>Pollen magnetofection to fruit harvest: 3 months;	1. Recalcitrance of mandarins<br>2. Difficulty in rooting of shoots and embryos<br>3. More than 10 years to flowering<br>4. Low regeneration competence<br>5. Necrosis from overgrowth<br>6. Concerns about foreign DNA<br>7. Long juvenility hindering segregation	Gabriela C ,Beatriz C X ,Gabriel P M , et al.Citrus Genetic Transformation: An Overview of the Current Strategies and Insights on the New Emerging Technologies [J].Frontiers in Plant Science,2021,12768197-	2021	Bush
15	*Acer truncatum* 'Lihong'	Acer, Sapindaceae	Method: Agrobacterium-mediated<br>Receptor material: Embryogenic callus<br>Agrobacterium strain: GV3101<br>Vector: pCAMBIA1305.1-GFP<br>Reporter gene: GFP<br>Selectable marker: Hygromycin<br>Bacterial density: OD600= 0.6–0.8<br>Infection time: 10 min<br>Acetosyringone (AS): 100 μmol/L<br>Co-cultivation time: 48 h<br>Co-cultivation temperature: 25°C<br>Co-cultivation light: Dark<br>Antibiotics for suppression: Carbenicillin200–300 mg/L+Cephalosporin 200 mg/L<br>Selection concentration: Hygromycin 25 mg/L<br>Best callus induction medium: 1/2MS + 2 mg/L 2,4-D + 0.3 mg/L 6-BA + 0.5 mg/L NAA<br>Best EC induction medium: 1/2MS + 3.0 mg/L 6-BA + 2.0 mg/L TDZ + 0.5 mg/L IBA + 0.1 mg/L GA_3<br>Best proliferation medium: WPM + 1.0 mg/L TDZ + 0.5 mg/L IBA + 0.1 mg/L GA_3 + 3 mg/L 6-BA + 1.0 mg/L KT	Callus induction rate: 100% (sterile seedling stem segment);<br>Embryogenic callus induction survival rate: 96.05%;<br>Embryogenic callus proliferation survival rate: 97.95%;<br>Proliferation coefficient: 5.12;<br>Optimal hygromycin selection concentration: 25 mg/L (survival rate >60%).	Callus induction, Embryogenic callus proliferation: 4 weeks;<br>Pre-culture: 1 day;<br>Infection: 10 min;<br>Co-cultivation: 48 h;<br>Antibacterial culture: 15 days;<br>Resistance screening: 7 days;<br>Total (explant to transgenic callus): Approximately 3–4 months.	1.High phenolic content and severe browning of outdoor materials<br>2.Only green granular callus among six types is embryogenic<br>3.Death after four months of subculture<br>4.Browning and decreased vigor after co-cultivation<br>5.Failure to achieve redifferentiation<br>6.False positives from low hygromycin; death from high concentration<br>7.Death from Agrobacterium encapsulation	Yang Y ,Chan Y ,Wang Y , et al.Preliminary establishment of genetic transformation system for embryogenic callus of Acer truncatum 'Lihong'[J].Frontiers in Plant Science,2024,151419313-1419313.DOI:10.3389/FPLS.2024.1419313.	2024	Arbor

16	*Citrus*	Citrus, Rutaceae	Method: Agrobacterium-mediated<br>Best strain: 1D1416 (A. fabrum)<br>Explant: Epicotyls (Carrizo), internodes (other cultivars)<br>Bacterial density: OD600 = 0.2–0.4<br>Infection time: 10–15 min<br>Acetosyringone: 200 µM (INM), 0.29 g/L (co-culture)<br>Co-cultivation time: 2–4 days<br>Co-cultivation temperature: 24°C<br>Co-cultivation light: Dark<br>Suppression antibiotics: Vancomycin 300 mg/L + Cefotaxime 350 mg/L<br>Selection antibiotic: Kanamycin 70 mg/L<br>Reporter gene: DsRed (red fluorescence)<br>Transient efficiency: 88% (1D1416 + surfactant)<br>Shoot regeneration efficiency: 42% (1D1416 + surfactant)	Best strain: 1D1416 (A. fabrum);<br>Transient efficiency (1D1416 + surfactant): 88%;<br>Gall formation (1D1416 + surfactant): 78%;<br>Transgenic shoot regeneration (1D1416 + surfactant): 42%;<br>EHA105 transient efficiency (with surfactant): 84%;<br>EHA105 shoot regeneration (with surfactant): 32%;<br>Mexican lime gall formation: 71%;<br>Optimal selection concentration: Kanamycin 70 mg/L;<br>DsRed expression in transgenic shoots: 79%.	Seed germination: 4 weeks;<br>Infection: 10–15 min;<br>Co-cultivation: 2–4 days;<br>SRM1 (dark): 14–21 days;<br>SRM2 (light): 14–21 days;<br>Total SRM2: 6–8 weeks;<br>Shoot maintenance: 3–4 weeks<br>Rooting: 2–3 weeks;<br>Total: Approximately 3–4 months.	1.Low efficiency with high necrosis<br>2.Only 5 of 41 successfully transforming citrus<br>3.Large variation among cultivars<br>4.Transfer of binary vector backbone<br>5.Only 69% identity with C58, with a unique T-DNA structure	Alabed D ,Tibebu R ,Ariyaratne M , et al.Novel Agrobacterium fabrum str. 1D1416 for Citrus Transformation[J].Microorganisms,2024,12(10):1999-1999.DOI:10.3390/MICROORGANISMS12101999.	2024	Bush
17	*Betula platyphylla*	Betula, Betulaceae	Method: CPDAT (Agrobacterium-mediated transient transformation)<br>Agrobacterium strain: EHA105<br>Vector: pYLCRISPR/Cas9P35S-N<br>Target gene: BpEBP1<br>Sonication: 24 W, 46 KHz, 15 s<br>Bacterial density: OD600= 0.7<br>Infection time: 2 h<br>Acetosyringone (AS): 120 µM<br>Suppression antibiotics: Cefotaxime 300 mg/L<br>Shoot induction medium: WPM + 1 mg/L 6-BA<br>Rooting medium: WPM + 0.2 mg/L NAA	Overall mutation rate: 80.00%;<br>Both alleles mutated: 40.00%;<br>Homozygous mutation rate: 23.08%;<br>Biallelic mutation rate: 13.85%;<br>DNA-free editing rate: 7.69% .	gRNA target screening: 4 days;<br>Transient transformation: 2 days;<br>Heat treatment & recovery: 10 days;<br>Adventitious bud induction: 30–40 days;<br>Plantlet growth: 30 days;<br>Mutation detection: 2–4 days;<br>DNA-free screening: 1 day;<br>Total: Approximately 2–3 months.	1.Low efficiency<br>2.Tendency of T-DNA integration<br>3.Low differentiation without selection pressure	Sun S ,Han X ,Jin R , et al.Generation of CRISPR-edited birch plants without DNA integration using Agrobacterium-mediated transformation technology.[J].Plant science : an international journal of experimental plant biology,2024,342112029-112029.DOI:10.1016/J.PLANTSCI.2024.112029.	2024	Arbor
18	*Actinidia spp.*	Actinidia, Actinidiaceae	Method: A. rhizogenes-mediated,A. tumefaciens<br>Strain: K599,EHA105<br>Explant: Leaves, hypocotyls, terminal buds,Petioles<br>Cultivar: A. latifolia 'Kuoye',A. eriantha 'White'<br>Bacterial density: OD600= 0.6–0.8,OD600= 0.6–0.8<br>Infection time: 8–10 min<br>Acetosyringone: 100 µM<br>Co-cultivation time: 2 days<br>Suppression antibiotics: Cefotaxime 300 mg/L,Timentin 300 mg/L<br>Selection antibiotics: Hygromycin 40 mg/L<br>Reporter gene: GFP<br>Efficiency improvement: 20% → 60%,26% → 40%<br>Positive rate improvement: 60% → 80%,40% → 50%	A.latifolia hairy root induction rate: 77% increase;<br>A. latifolia hairy root regeneration: <20% → 60%;<br>A. latifolia positive rate: 60% → 80%;<br>A. latifolia callus induction: >80%;<br>A. erANtha regeneration efficiency: 26% → 40%;<br>A. eriantha positive rate: 40% → 50%.	A. Rhizogenes:<br>Explant preparation: 4–5 weeks;<br>Infection: 8–10 min;<br>Co-cultivation: 2 days;<br>Hairy root induction: 5–6 weeks;<br>Callus and regeneration: 6 weeks;<br>Total: 2–3 months;<br>A.tumefaciens:<br>Explant preparation: 4–5 weeks;<br>Infection: 8–10 min;<br>Co-cultivation: 2 days;<br>Selection & regeneration:<br>Replace every 15 days;<br>Total: 2–3 months.	1.Species limitation: Non-transformability of A. latifolia<br>2.Regeneration efficiency: Hairy root regeneration rate below 20%<br>3.Gene expression: Low expression of AIGRF4 and AIGIF1<br>4.Transformation cycle: Transformation cycle exceeding 6–8 months in woody plants<br>5.DR side effects: Developmental abnormalities caused by WUS and BBM<br>6.Genotype dependence: Methods applicability limited to specific genotypes	He Y ,Bai Z ,Wu T , et al.AcGRF4-AcGIF1 chimeric protein improves the transformation and regeneration efficiency in kiwifruit[J].Scientia Horticulturae,2025,354114518-114518.DOI:10.1016/J.SCIENTA.2025.114518.	2025	Woody Vine
19	*Eremosparton songoricum* (Litv.) Vass.	Eremosparton, Fabaceae	Method: Agrobacterium-mediated transient transformation<br>Reporter gene: GUS<br>Best strain for seedlings: GV3101<br>Best strain for assimilated branches: C58C1<br>Optimal OD600 for seedlings: 0.6<br>Optimal OD600 for assimilated branches: 0.8<br>Acetosyringone (AS): 200 µM<br>Sucrose concentration: 3%<br>Infection time: 3 h<br>Co-cultivation time: 2 days (dark)<br>Base medium: 1/2 MS<br>Callus transformation solution: (AB: MES): MS = 1: 1<br>Highest efficiency (assimilated branches): 97%<br>Seedling efficiency: 57.17%<br>Callus efficiency: 39.51%	Seedling highest efficiency: 57.17%;<br>Assimilated branch highest efficiency: 97%;<br>Callus highest efficiency: 39.51%.	Seed germination: 3–13 days<br>Infection: 3 h<br>Co-cultivation: 2 days<br>Peak expression: 2 days<br>Expression duration: 10 days<br>Total experimental period: 2–3 weeks	1.Absence of an established transformation system<br>2.Differential responses among explant types<br>3.Impact of Seedling age: extremes problematic<br>4.High phenolic content leading to browning<br>5.Strain specificity required for different explants<br>6.Ineffectiveness of conventional solutions for callus	Lao X ,Jin P ,Yang R , et al.Establishment of Agrobacterium-Mediated Transient Transformation System in Desert Legume Eremosparton songoricum (Litv.) Vass.[J].International journal of molecular sciences,2024,25(22):DOI:10.3390/IJMS252211934.	2024	Woody Plant

20	*Capsicum annuum* L.	Capsicum, Solanaceae	Method: Agrobacterium-mediated<br>Agrobacterium strain: EHA105<br>Explant: Cotyledons, hypocotyls, leaves (roots ineffective)<br>Best varieties: CM334,Zunla-1<br>Pre-culture time: 24 h<br>Bacterial density: OD600 = 0.1<br>Infection time: 15–30 min<br>Co-cultivation time: 72 h<br>Acetosyringone: 20 mg/L<br>Suppression antibiotic: Cefotaxime 300 mg/L<br>Selection antibiotic: Hygromycin 30 mg/L<br>Optimal shoot induction hormones: 5 mg/L BAP + 1 mg/L IAA + 5 mg/L $AgNO_3$ or 4 mg/L TDZ + 1 mg/L IAA + 5 mg/L $AgNO_3$<br>Optimal GA_3 for elongation: 0.5 mg/L<br>Optimal IBA for rooting: 1 mg/L<br>Highest regeneration rate: 41% (CM334 cotyledons)<br>Highest transformation efficiency: 3.5% (CM334 leaf, pGH-rGRF-GIF)	Highest regeneration rate: 41% (CM334 cotyledons);<br>Highest transformation efficiency: 3.5% (CM334 leaf, pGH-rGRF-GIF);<br>Second highest transformation efficiency: 2.2% (Zunla-1 leaf, pGH-rGRF-GIF).	Seed germination: 2–3 weeks;<br>Callus induction: 36–48 days;<br>Shoot induction: 30–80 days (varies by variety);<br>Shoot elongation: 60–90 days;<br>Rooting: 15–20 days;<br>Water acclimatization: 1 week;<br>Greenhouse growth: 3–4 months;<br>Pre-culture: 24 h;<br>Infection: 15–30 min;<br>Co-cultivation: 72 h;<br>Selection and regeneration: 2 months;<br>Total transformation timeline: Approximately 2–3 months (from infection to transgenic plants);<br>Total regeneration timeline: Approximately 3–4 months (from seed to complete plants).	1.Genotype dependence: lack of shoot formation in 4 out of 7 varieties<br>2.Explant selection: ineffectiveness of root explants<br>3.Regeneration efficiency: maximum of 41%<br>4.Transformation efficiency: maximum of 3.5%<br>5.Tissue necrosis: prolonged co-cultivation or high density leading to necrosis<br>6.Suppression antibiotic: difficulty in controlling cefotaxime concentration<br>7.Selection pressure: Difficulty in controlling hygromycin concentration	Shams S ,Naeem B ,Ma L , et al.Developing an Optimized Protocol for Regeneration and Transformation in Pepper[J].Genes,2024,15(8):1018-1018.DOI:10.3390/GENES15081018.	2024	Suffrutescent Plant
